# Near-infrared absorbing acceptor with suppressed triplet exciton generation enabling high performance tandem organic solar cells

**DOI:** 10.1038/s41467-023-36917-y

**Published:** 2023-03-04

**Authors:** Zhenrong Jia, Qing Ma, Zeng Chen, Lei Meng, Nakul Jain, Indunil Angunawela, Shucheng Qin, Xiaolei Kong, Xiaojun Li, Yang (Michael) Yang, Haiming Zhu, Harald Ade, Feng Gao, Yongfang Li

**Affiliations:** 1 Beijing National Laboratory for Molecular Sciences, CAS Key Laboratory of Organic Solids, Institute of Chemistry, Chinese Academy of Sciences, Beijing, 100190 China; 2 School of Chemical Science, University of Chinese Academy of Sciences, Beijing, 100049 China; 3 State Key Laboratory of Modern Optical Instrumentation, Key Laboratory of Excited State Materials of Zhejiang Province, Department of Chemistry, Zhejiang University, Hangzhou, Zhejiang 310027 China; 4 State Key Laboratory of Modern Optical Instrumentation, College of Optical Science and Engineering, Zhejiang University, Hangzhou, Zhejiang 310027 China; 5 Department of Physics, Chemistry, and Biology (IFM), Linköping University, Linköping, SE-58183 Sweden; 6 Department of Physics and Organic and Carbon Electronics Laboratories (ORaCEL), North Carolina State University, Raleigh, NC 27695 USA; 7 Laboratory of Advanced Optoelectronic Materials, Suzhou Key Laboratory of Novel Semiconductor-optoelectronics Materials and Devices, College of Chemistry, Chemical Engineering and Materials Science, Soochow University, Suzhou, Jiangsu 215123 China

**Keywords:** Solar cells, Solar cells

## Abstract

Reducing the energy loss of sub-cells is critical for high performance tandem organic solar cells, while it is limited by the severe non-radiative voltage loss via the formation of non-emissive triplet excitons. Herein, we develop an ultra-narrow bandgap acceptor BTPSeV-4F through replacement of terminal thiophene by selenophene in the central fused ring of BTPSV-4F, for constructing efficient tandem organic solar cells. The selenophene substitution further decrease the optical bandgap of BTPSV-4F to 1.17 eV and suppress the formation of triplet exciton in the BTPSV-4F-based devices. The organic solar cells with BTPSeV-4F as acceptor demonstrate a higher power conversion efficiency of 14.2% with a record high short-circuit current density of 30.1 mA cm^−2^ and low energy loss of 0.55 eV benefitted from the low non-radiative energy loss due to the suppression of triplet exciton formation. We also develop a high-performance medium bandgap acceptor O1-Br for front cells. By integrating the PM6:O1-Br based front cells with the PTB7-Th:BTPSeV-4F based rear cells, the tandem organic solar cell demonstrates a power conversion efficiency of 19%. The results indicate that the suppression of triplet excitons formation in the near-infrared-absorbing acceptor by molecular design is an effective way to improve the photovoltaic performance of the tandem organic solar cells.

## Abstract (web summary)

Reducing energy loss of sub-cells is critical for high performance tandem organic solar cells. Here, the authors design and synthesize an ultra-narrow bandgap acceptor through replacement of terminal thiophene by selenophene in the central fused ring, achieving efficiency of 19% for tandem cells.

## Introduction

Tandem organic solar cell (TOSC) that pairs photoactive layers with complementary absorption is a promising technique for surpassing the efficiency limit of single junction OSCs^1–6^. In the series-connected TOSCs, the high energy photons are utilized to generate a high open circuit voltage (*V*_oc_) in the front subcell while the lower energy photons are absorbed in the rear subcell to get a balanced short-circuit current density (*J*_sc_). Such strategies can effectively reduce the thermalization loss of photon energy and broaden overall absorption spectrum utilization compared to single junction devices^7–10^. In recent years, small molecule acceptors (SMAs) have shown great tunability in optical bandgap (*E*_g_^opt^) and electron energy levels, providing a wide range of new opportunities for building highly efficient TOSCs^11–15^. Great efforts have been devoted to developing wide bandgap (WBG) and narrow bandgap (NBG) acceptors and great progress has been made in fabricating high-performance TOSCs^16–19^.

Yang et al. adopted mixed narrow bandgap acceptors F8IC and FOIC as the rear cell materials and achieved NREL certified power conversion efficiency (PCE) of 11.6% in 2019^20^. To further broaden the absorption, Chen et al. adopted O6T-4F with infrared absorption edge at 1050 nm as the rear cell material and achieved the TOSC with PCE of 17.3%^21^. To decrease the energy loss of front cell, Hou et al. synthesized a wide bandgap non-fused acceptor GS-ISO, and the tandem organic photovoltaic cell adopting PBDB-TF:GS-ISO as the front subcell showed PCE of 19–20%^22,23^.

From the device modeling based on Shockley-Queisser (SQ) limit analysis, there is still much room for promoting power conversion efficiency (PCE) of the TOSCs^24,25^. At first, the active layer of the rear cell should have an optimal bandgap of ∼1.1–1.2 eV^21^. Although a few ultra-narrow bandgap acceptors could meet the demand of *E*_g_^opt^, it is still difficult to simultaneously offer a high *J*_sc_ with high external quantum efficiency (EQE) response in the near-infrared (NIR) region^25–28^. Moreover, in the condensed phase, molecules with emission energy gaps in the near-infrared (NIR) region are expected to yield much lower emission intensities, which has long been recognized as the Energy Gap Law^29–31^. As governed by the Energy Gap Law, the non-radiative voltage losses could be increased with decreasing charge-transfer-state energies^32–36^. In the donor:acceptor (D:A) based blend films in OSCs, the lower emission intensity in the NIR region is specifically caused by the commonly encountered energy transition between the triplet (T_1_) excited state and the ground state (S_0_), and this dynamics is more dominating in the ultra-NBG photovoltaic materials due to the higher overlap of the wavefunctions of S_1_ or T_1_ states and the higher isoenergetic vibration of the S_0_ state^30,37,38^. The fast non-radiative transition pathway from T_1_ to S_0_ could significantly reduce the emission intensity, which causes the higher recombination loss^39,40^.

Inspired by recent advances of A-DA’D-A acceptors based single junction devices^14,41^, it is a promising strategy to design A-DA’D-A type SMAs with suitable bandgaps for constructing complementary absorbing sub-cells of TOSCs. In this work, we designed and synthesized a new ultra-narrow bandgap SMA BTPSeV-4F through replacement of terminal thiophene by selenophene in the central fused ring of the NBG SMA BTPSV-4F^42^. In comparison with BTPSV-4F, BTPSeV-4F shows further red-shifted absorption with absorption edge at 1061 nm corresponding to an *E*_g_^opt^ of as low as 1.17 eV. The single-junction OSCs based on BTPSeV-4F as acceptor and PTB7-Th as polymer donor demonstrated a higher PCE of 14.2% with a record high *J*_sc_ of 30.1 mA cm^−2^, which is the highest-performed OSC based on NIR acceptor with optical bandgaps below 1.2 eV so far. In addition, for better bandgap matching with NBG SMAs in the TOSC, we further designed and synthesized a medium bandgap A-DA′D-A acceptor O1-Br as the SMA of the front cell through bandgap engineering toward the higher *E*_g_^opt^ of 1.58 eV. O1-Br shows over 100 nm blue-shifted absorption spectrum with the absorption edge of 786 nm in comparison with Y6. With BTPSeV-4F and O1-Br as the acceptor materials in rear cell and front cell respectively, the resulting TOSCs reached a high PCE of 19.0%.

## Results

### Material design and characterization

**Fig. 1:**
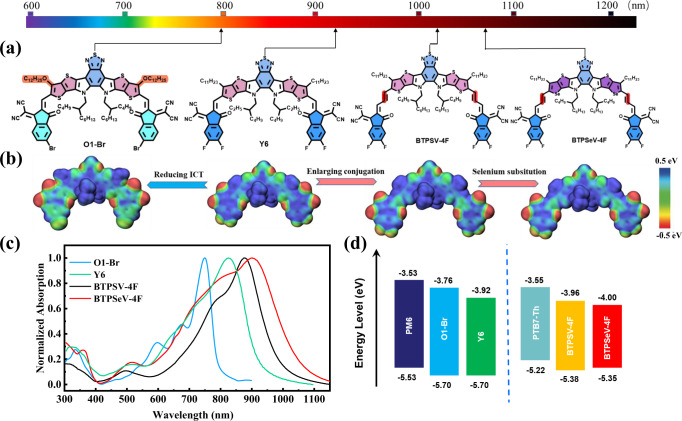
Materials design and characterization. **a** Molecular structures of the acceptors with arrows indicating absorption edge wavelength of the acceptor films. **b** ESP distributions for the simplified structures of O1-Br, Y6, BTPSV-4F, and BTPSeV-4F. **c** Absorption spectra of the O1-Br, Y6, BTPSV-4F, and BTPSeV-4F films. Source data are provided as a Source Data file. **d** Energy level diagram of the donors and acceptors.

Considering that the A-DA’D-A structured SMAs led the breakthrough in the single-junction OSCs, we further designed the acceptors with suitable *E*_g_^opt^ for the application in the front cell and rear cell of the TOSCs based on the A-DA’D-A structured SMAs. Figure 1a shows the chemical structures of acceptors, including our designed medium bandgap O1-Br and ultra-narrow bandgap BTPSeV-4F for the application as acceptors in front and rear cell of the TOSCs, and the representative SMAs of narrow bandgap Y6 and ultra-narrow bandgap BTPSV-4F from which the new acceptors were designed. The detailed synthetic processes and characterizations of the new SMAs BTPSeV-4F and O1-Br are described in the “Methods” section. BTPSeV-4F was designed and synthesized by the replacement of terminal thiophene with selenophene in the central fused ring of BTPSV-4F^37^. The reduced aromaticity of selenophene could enhance the ground-state quinoidal resonance character to result in narrower *E*_g_^opt^ of BTPSeV-4F^43,44^. The absorption spectrum of BTPSeV-4F film shows a significant red-shift in comparison with BTPSV-4F film (Fig. 1c), with the absorption edge at 1061 nm corresponding to an *E*_g_^opt^ of 1.17 eV.

For fabricating high performance TOSCs, it is also important to design the medium bandgap acceptor for the front cell. In order to simultaneously get ideal bandgap and higher *V*_oc_, a new medium bandgap acceptor O1-Br was designed and synthesized by modification of Y6 with introducing monobromine substitution instead of bifluorine substitution on the end group and replacing the upper alkyl side chains with alkoxy side chains^45,46^ for blue-shifting its absorption. As expected, the absorption spectrum of O1-Br film is blue-shifted with an *E*_g_^opt^ of 1.58 eV (as shown in the Fig. 1c). Supplementary Figure 1 shows the absorption spectra of the acceptors in chloroform solutions.

To verify the effects of above-mentioned strategies in the molecular design of the acceptors, we performed theoretical calculation by using density functional theory (DFT). Figure 1b shows the electrostatic potential (ESP) distributions of the four acceptors. Electrostatic surface potentials are colored red and blue for negative and positive charges, respectively. With the introduction of two double bonds, conjugation length of BTPSV-4F and BTPSeV-4F can effectively extended, forming larger π conjugated systems. In addition, the intermolecular interactions are significantly related to the ESP distributions on the molecular surfaces. Obviously, ESP values on the IC terminal of O1-Br are much lower than those in Y6, indicating that introducing monobromine substitution and alkoxy side chains can effectively increase the electronic density of the terminal moieties thus reduce the ICT effect of acceptors. Moreover, simulated molecular geometries are also displayed in Supplementary Fig. 2. All the two newly designed electron acceptors exhibit almost similar conformation with Y6, which is propitious to form efficient charge transport channel. The calculated lowest unoccupied molecular orbital (LUMO) and the highest occupied molecular orbital (HOMO) electronic density distributions of the four acceptors were shown in Supplementary Fig. 2. The result reveals the obviously narrower bandgap of BTPSeV-4F and the wider bandgap of O1-Br.

Cyclic voltammetry measurements were performed to estimate electronic energy levels of the acceptors with Ag/AgCl as the reference electrode^47^, and the measured cyclic voltammograms are shown in Supplementary Fig. 3 where the onset oxidation/reduction potentials (φ_ox/red_) can be obtained. Then the HOMO energy level (*E*_HOMO_) and LUMO energy level (*E*_LUMO_) were calculated according to the following Eq. (1):

1 $${E}_{{{{{{\rm{HOMO}}}}}}/{{{{{\rm{LUMO}}}}}}}=-\!{{{{{\rm{e}}}}}}(4.80+{\varphi }_{{{{{{\rm{ox}}}}}}/{{{{{\rm{red}}}}}}}-{\varphi }_{{{{{{\rm{Fc}}}}}}+/{{{{{\rm{Fc}}}}}}})$$

where the redox potential φ_Fc+/Fc_ was measured to be 0.44 V vs. Ag/AgCl in our measurement system, and the calculated *E*_HOMO_ and *E*_LUMO_ values of the acceptors were listed in Supplementary Table 1. In comparison with BTPSV-4F, the *E*_HOMO_ of BTPSeV-4F is upshifted from −5.38 to −5.36 eV and its *E*_LUMO_ is down-shifted from −3.96 to −4.00 eV, which agrees with the red-shifted absorption of BTPSeV-4F. While for O1-Br in comparison with Y6, its *E*_LUMO_ is upshifted from −3.92 to −3.76 eV with the same *E*_HOMO_ of −5.70 eV, which agrees with the blue-shift of its absorption, and the upshifted *E*_LUMO_ of the acceptor is beneficial to higher *V*_oc_ of the OSCs. In considering the complementary absorption and energy levels matching of the donor and acceptor materials (see Fig. 1d), we selected the wide bandgap polymer PM6 as the donor for the front cell with O1-Br as acceptor and the narrow bandgap polymer PTB7-Th as the donor for the rear cell with BTPSeV-4F as acceptor.

### Photovoltaic performance of BTPSeV-4F

**Fig. 2:**
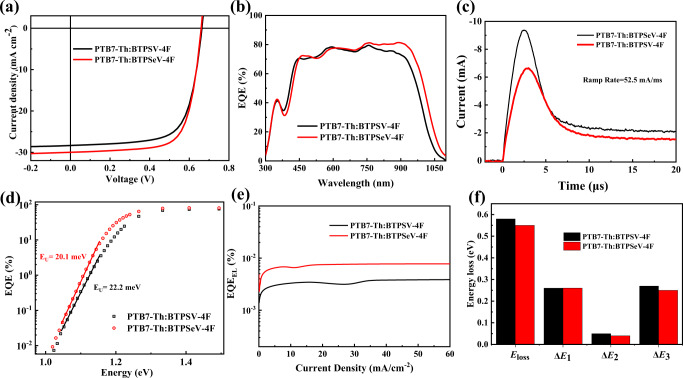
Photovoltaic performance of ultra-narrow bandgap acceptors. **a** *J*−*V* curves of the optimized OSCs based on PTB7-Th:BTPSV-4F and PTB7-Th:BTPSeV-4F, under the illumination of AM1.5G, 100 mW cm^−2^. Source data are provided as a Source Data file. **b** The external quantum efficiency (EQE) spectra of the corresponding OSCs. Source data are provided as a Source Data file. **c** The Photo-CELIV curves of the corresponding OSCs. Source data are provided as a Source Data file. **d** FTPS-EQE curves of the corresponding OSCs. Source data are provided as a Source Data file. **e** The external electroluminescence quantum efficiency (EQE_EL_) of the devices. Source data are provided as a Source Data file. **f** Summary of *E*_loss_ analysis of the corresponding OSCs. Source data are provided as a Source Data file.

To check the photovoltaic properties of OSCs based on BTPSV-4F and BTPSeV-4F for the application as rear cell in TOSCs, PTB7-Th was selected as the polymer donor with a conventional device structure of indium tin oxide (ITO)/PEDOT:PSS/active layer/PDINN/Ag. The fabrication conditions of the OSCs were optimized and the results are listed in Supplementary Table 2. Figure 2a shows the current density*–*voltage (*J*–*V*) curves of the optimized devices under the illumination of AM1.5G, 100 mW cm^−^^2^, and the detailed photovoltaic parameters are listed in Table 1. The OSC based on PTB7-Th:BTPSV-4F shows a PCE of 13.0% with a *V*_oc_ of 0.66 V, *J*_sc_ of 28.4 mA cm^−^^2^ and an FF of 69.5%. Encouragingly, the PTB7-Th:BTPSeV-4F based OSC achieved a better PCE of 14.2% with *V*_oc_ of 0.660 V, a higher *J*_sc_ of 30.1 mA cm^−^^2^ and FF of 71.4%. Notably, 30.1 mA cm^−^^2^ is the highest *J*_sc_ value reported to date in the field of OSCs. The enhanced *J*_sc_ resulted from the obviously red-shifted absorption of BTPSeV-4F and the better FF could be due to enhanced intermolecular Se−Se interaction^39^ and increased charge carrier mobility. The external quantum efficiency (EQE) spectra of the OSCs based on PTB7-Th:BTPSV-4F and PTB7-Th:BTPSeV-4F were measured and shown in Fig. 2b. Both OSC devices show broad and strong photoresponse in the NIR region. Benefitted from the narrower *E*_g_^opt^ and stronger photoresponse in the NIR region of the BTPSeV-4F based devices, the integrated *J*_sc_ from the EQE values reached 27.76 and 29.30 mA cm^−2^ for the OSCs based on BTPSV-4F and BTPSeV-4F, respectively, which is in good agreement with the *J*_sc_ values measured from the *J*–*V* curves. It is worth noting that the BTPSeV-4F based device shows obviously higher EQE response in the long wavelength range, corresponding to the extended optical absorption of BTPSeV-4F, as well as the desirable charge-transporting properties induced by enhanced intermolecular Se–Se interaction. The relationship between photocurrent density (*J*_ph_) and effective voltage (*V*_eff_) of the OSCs were further characterized to study the exciton dissociation behavior of the devices. As shown in Supplementary Fig. 4, the *J*_ph_ /*J*_sat_ ratios are determined to be 0.95 and 0.97 for the BTPSV-4F and BTPSeV-4F based OSCs, respectively, indicating that the BTPSeV-4F-based device has more efficient exciton dissociation efficiencies, which is one of responsibility for its high *J*_sc_.

**Table 1:** Photovoltaic parameters of the optimal single-junction OSCs based on PTB7-Th:BTPSV-4F and PTB7-Th:BTPSeV-4F, under the illumination of AM 1.5G, 100 mW cm^−2^

Device	*V*_oc_ [V]	*J*_sc_ [mA·cm^−^^2^]	*J*_cal_ from EQE [mA·cm^−^^2^]	FF [%]	PCE [%]
PTB7-Th:BTPSV-4F	0.66 (0.66 ± 0.01)	28.4 (28.1 ± 0.3)	27.76	69.5 (69.2 ± 0.6)	13.0 (12.8 ± 0.2)^a^
PTB7-Th:BTPSeV-4F	0.66 (0.66 ± 0.01)	30.1 (29.8 ± 0.2)	29.30	71.4 (71.0 ± 0.4)	14.2 (13.9 ± 0.2)

^a^The average values and deviations are calculated from 10 independent devices.

To understand the charge transport behavior in the OSCs based on BTPSV-4F and BTPSeV-4F, we carried out the measurements of photo-induced charge-carrier extraction in linearly increasing voltage (photo-CELIV)^48^. Figure 2c shows the transient signal of photo-CELIV in the BTPSV-4F and BTPSeV-4F-based devices. And the carrier extraction mobility in photo-CELIV was measured to be 1.46 × 10^–4^ and 1.99 ×10^–4^ cm^2^ V^–1^ s^–1^ for the devices based on PTB7-Th:BTPSV-4F and PTB7-Th:BTPSeV-4F, respectively.

Furthermore, the electron mobility (*µ*_e_) and hole mobility (*µ*_h_) of the films of BTPSV-4F, BTPSeV-4F, PTB7-Th:BTPSV-4F and PTB7-Th:BTPSeV-4F were estimated using the space charge limited current (SCLC) method. As expected, the *µ*_e_ of BTPSeV-4F (4.2 × 10^–4^ cm^2^ V^–1^ s^–1^) is higher than that of BTPSV-4F (3.5 × 10^–4^ cm^2^ V^–1^ s^–1^), as shown in Supplementary Fig. 5 and Supplementary Table 3. In Supplementary Fig. 6, the *µ*_h_ and *µ*_e_ values of PTB7-Th:BTPSV-4F films are estimated to be 2.3 × 10^−^^4^ cm^2^ V^−^^1^ s^−^^1^ and 2.5 × 10^–4^ cm^2^ V^−1^ s^−^^1^, respectively. As for the PTB7-Th:BTPSeV-4F films, the corresponding *µ*_h_ and *µ*_e_ values are 3.6 × 10^–4^ cm^2^ V^−^^1^ s^−^^1^ and 4.1 × 10^–4^ cm^2^ V^−^^1^ s^−^^1^ respectively. The increased electron mobilities in the PTB7-Th:BTPSeV-4F blends could be accountable for the higher FF in the OSCs.

As governed by the Energy Gap Law, the non-radiative voltage losses could be increased with decreasing charge-transfer-state energies. Therefore, the development of high-performance ultra-narrow bandgap OSC is often hindered by strong non-radiative process and suffers low *V*_oc_ in constructing TOSCs^29^. Surprisingly, despite BTPSeV-4F shows narrower *E*_g_^opt^, the *V*_oc_ of BTPSV-4F and BTPSeV-4F based OSCs are quite similar. To investigate the reasons behind the unusual phenomenon of the *V*_oc_, we studied the energy losses in both devices. The total voltage loss can be calculated by the following equation:^49^

2 $${E}_{{{{{\rm{loss}}}}}}=	 {E}_{g}-q{V}_{{{{{\rm{oc}}}}}}\\ \,=	 ({E}_{g}-q{V}_{oc}^{SQ})+(q{V}_{OC}^{SQ}-q{V}_{OC}^{{{{{\rm{rad}}}}}})+(q{V}_{OC}^{{{{{\rm{rad}}}}}}-q{V}_{OC})\\ \,=	 \varDelta {E}_{1}+\varDelta {E}_{2}+\varDelta {E}_{3},$$

where *E*_g_ is the optical bandgap of the corresponding active layers, *q* is the elementary charge, Δ*E*_1_ is the radiative loss above bandgap, Δ*E*_2_ is the radiative loss below bandgap, and Δ*E*_3_ is the nonradiative loss. The Δ*E*_3_ is equal to *q*Δ*V*_nr_, and Δ*V*_nr_ is the non-radiative recombination voltage loss, which can be calculated from the EQE_EL_ of the devices by the equation of

3 $$\varDelta {V}_{nr}=\frac{kT}{q}\,{{{{\mathrm{ln}}}}}\left(\frac{1}{EQ{E}_{EL}}\right)$$

where *k* is Boltzmann constant and *T* is Kelvin temperature^50^. The specific energy losses of the devices based on PTB7-Th:BTPSV-4F and PTB7-Th:BTPSeV-4F were summarized in Supplementary Table 4. As shown in Supplementary Fig. 7, the *E*_g_ of the blend films of PTB7-Th:BTPSV-4F and PTB7-Th:BTPSeV-4F were 1.25 and 1.22 eV, respectively. The optical bandgap of the device is calculated by considering the derivative of the EQE spectrum^50^. For any types of solar cells, Δ*E*_1_ is unavoidable. Herein, the BTPSV-4F and BTPSeV-4F based OSCs showed the almost same Δ*E*_1_ value (~0.27 eV). For Δ*E*_2_, the BTPSeV-4F based device shows slightly smaller Δ*E*_2_ (0.04 eV) than the BTPSV-4F based device (0.05 eV), as shown in Fig. 2d. More importantly, the EQE_EL_ value of the BTPSeV-4F based device (7.4 × 10^−5^) is much higher than that of the BTPSV-4F based device (3.2 × 10^−^^5^), as shown in Fig. 2e. Thus, the BTPSeV-4F based device exhibits a reduced non-radiative recombination loss Δ*E*_3_ (0.25 eV) compared to that of the BTPSV-4F based device (0.27 eV). The component of non-radiative energy loss should be the major contribution to the decreased energy loss in the PTB7-Th:BTPSeV-4F based device (Fig. 2f).

In addition, we also analyzed Urbach energy (*E*_u_) of the two blends from Fourier transform photocurrent spectroscopy EQE (FTPS-EQE) spectra^51^. The value of *E*_u_ can be quantified by equation of $\alpha \left(E\right)={\alpha }_{0}{e}^{\frac{\left(E-{E}_{0}\right)}{{E}_{u}}}$, where $\alpha \left(E\right)$ is the absorption coefficient, ${\alpha }_{0}$ and ${E}_{0}$ are two constants, and *E* is the photon energy^52^. As shown in Fig. 2d, the blend films of PTB7-Th:BTPSeV-4F showed much lower *E*_u_ values of 20.1 meV, compared with that of 22.2 meV for PTB7-Th:BTPSV-4F. To the best of our knowledge, the Urbach energy of 20.1 meV for the PTB7-Th:BTPSeV-4F based device is the lowest value reported so far for OSCs^52^. The lower Urbach energy benefits the decrease of energetic disorder, thus reduces the radiative loss below bandgap and the non-radiative recombination loss^53,54^.

**Fig. 3:**
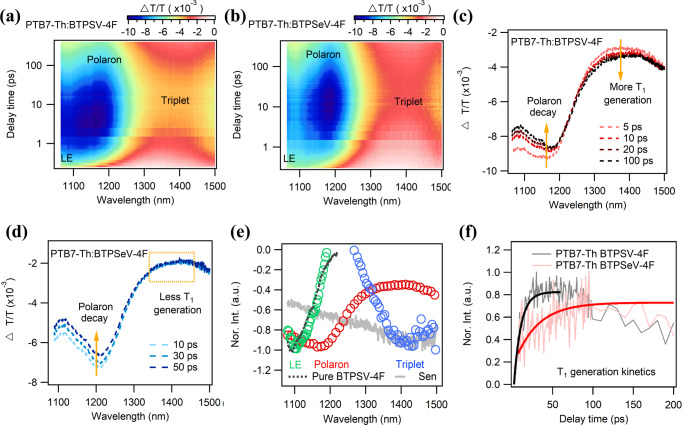
Triplet generation dynamics of blend films in NIR-TA spectra. **a**, **b** 2D color plot of TA spectra of PTB7-Th:BTPSV-4F film and PTB7-Th:BTPSeV-4F film under 900 nm excitation at ~10 μJ/cm^2^. **c** TA spectra of PTB7-Th:BTPSV-4F at the indicated delay times in NIR region. **d** TA spectra of PTB7-Th:BTPSeV-4F at the indicated delay times in NIR region. **e** Extracted characteristic TA spectra of BTPSV-4F. **f** The extracted triplet exciton formation kinetics in PTB7-Th:BTPSV-4F and PTB7-Th:BTPSeV-4F films and their single exponential fits.

To investigate the mechanism of the reduced non-radiative loss in the PTB7-Th:BTPSeV-4F -based device, we studied the charge carrier dynamics in the OSCs based on transient absorption (TA) spectroscopy in Vis-NIR region^37^. Here, we used a 900 nm laser (~10 μJ/cm^2^) to excite BTPSV-4F, BTPSeV-4F and their blends films. The 2D color TA spectra and a few representative TA spectra at selected delay times of pure BTPSV-4F and BTPSeV-4F films were displayed in Supplementary Fig. 8, showing the excited state absorption peak of both singlet excitons at ~1100 nm with slight red shift spectra in the BTPSeV-4F based films. Figure 3a–d presented the 2D color TA spectra and a few representative TA spectra at selected delay times of the PTB7-Th:BTPSV-4F and PTB7-Th:BTPSeV-4F films. The peak that appeared within 2 ps at ~1200 nm was ascribed to the polaron signal, which is in good agreement with PTB7-Th bleach generation process (i.e., photo-induced hole transfer process, see Supplementary Figs. 9, 10 in detail). Then, as shown in Fig. 3b, the polaron peak started to decay accompanied by a broad signal generation at around 1400 nm, matching well with the BTPSV-4F triplet sensitization TA spectra exhibited in Fig. 3e (gray line). Therefore, the signal rising at ~1400 nm can be explained by free carrier recombination to the triplet states of the narrow bandgap acceptors. Compared with PTB7-Th:BTPSV-4F, the rising signal at ~1400 nm seems not so obvious in the PTB7-Th:BTPSeV-4F blend film (see Fig. 3d), suggesting less triplet generation in PTB7-Th:BTPSeV-4F. To further investigate the triplet exciton generation dynamics, single value decomposition (SVD) was used to differentiate species and their kinetics in decomposition complex TA spectra evolution. Figure 3e exhibits three species obtained from PTB7-Th:BTPSV-4F by SVD with residuals ~0.1%, corresponding to the localized exciton, polaron and triplet exciton, respectively. The extracted triplet exciton generation kinetics for PTB7-Th:BTPSV-4F and PTB7-Th:BTPSeV-4F are compared in Fig. 3f. Clearly, the triplet exciton formation time of PTB7-Th:BTPSV-4F (12.7 ± 1.3 ps by single exponential fitting) is faster than that of PTB7-Th:BTPSeV-4F (31.6 ± 3.2 ps by single exponential fitting), re-confirming the reduced triplet exciton generation by free carrier recombination in the BTPSeV-4F based blend film. Since the formation of triplet excitons from the free carrier recombination is one of the main energy loss channels in OSCs, as shown in Supplementary Fig. 11^34^. These results could well explain the reduced non-radiative recombination loss in PTB7-Th:BTPSeV-4F, suggesting that the selenophene-substitution can inhibit charge recombination to triplet excitons in the acceptor.

To suppress charge recombination via the triplet channel, it is imperative to reduce the singlet−triplet energy gap (Δ*E*_ST_) of the narrow bandgap acceptors^55^. DFT calculations have also been performed on the two acceptors to characterize the nature of excited triplet states^56^. The frontier molecular orbitals of the acceptors are shown in Supplementary Fig. 12 with the oscillator strength (*f*), degree of the HOMO − LUMO overlap (Θ_H−L_) and the weights of the HOMO → LUMO transition in the S_1_ and T_1_ excitations. The LUMO is delocalized over the whole molecular backbone while the HOMO is localized mainly in the end group of the two acceptors. The Θ_H−L_ of molecules are calculated to be 63.4 and 62.0% for BTPSV-4F and BTPSeV-4F, respectively. The lower degree of the HOMO − LUMO overlap in BTPSeV-4F can increase the average distance between the photoexcited electron and hole pair, which is beneficial to reduce the singlet−triplet energy gap (Δ*E*_ST_)^57^. Moreover, the excitation energies of S_1_ and T_1_ and corresponding Δ*E*_ST_ are shown in Supplementary Fig. 13. The BTPSeV-4F acceptor exhibits a reduced Δ*E*_ST_ (0.22 eV) compared to that of the BTPSV-4F acceptor (0.25 eV). The reduction of Δ*E*_ST_ could contribute to mitigating the losses produced by the nonradiative deactivation of the T1 excitons^58^. These results theoretically support our molecular design, suggesting that the introduction of selenium could decrease Δ*E*_ST_ of molecules and thus suppress the triplet recombination processes.

In order to investigate the molecular packing properties of the active layer, grazing-incidence wide-angle X-ray scattering (GIWAXS) measurements were performed^59^ (https://iopscience.iop.org/article/10.1088/1742-6596/247/1/012007/meta). For the BTPSV-4F and BTPSeV-4F neat films (Supplementary Fig. 14 and Supplementary Table 5), the π–π stacking diffractions are located at 1.76 Å^−1^ for BTPSV-4F and 1.78 Å^−1^ for BTPSeV-4F. The π–π stacking distance (*d*) /CCL (crystalline coherence length) of these acceptors are 3.57/27 Å for BTPSV-4F and 3.53/21 Å for BTPSeV-4F. The normalized integrated intensity of the π-π staking is considerably higher in the BTPSeV-4F film than that in the BTPSV-4F film. The relatively higher diffraction intensity can be interpreted as a larger volume fraction of ordered molecules with π-π stacking, which can enhance charge transport. Both the diffraction intensity and coherence length have been previously shown to correlate well with device performance. By mixed with PTB7-Th polymer donor, the two blend films show a predominantly face-on orientation (Supplementary Fig. 15 and Supplementary Tables 6, 7). For the donor PTB7-Th, the π–π stacking distance in the PTB7-Th:BTPSeV-4F is 3.66 Å which is slightly shorter than 3.71 Å in the PTB7-Th:BTPSV-4F film. In the meanwhile, for the SMAs, PTB7-Th:BTPSV-4F and PTB7-Th:BTPSeV-4F films show similar π–π stacking distance. However, there is a longer CCL in the PTB7-Th:BTPSeV-4F blends (59 Å) compared to that in the PTB7-Th:BTPSV-4F blends (43 Å) blends. The PTB7-Th:BTPSeV-4F films also show much higher normalized integrated intensity. All the results indicate that replacing sulfur with selenium in the fused ring unit can effectively strengthen the inter-molecular interactions and improve molecular packing and charge transport properties of the acceptors^41^.

Furthermore, atomic force microscopy (AFM) and transmission electron microscopy (TEM) were measured to probe morphology of the active layers. As shown in Supplementary Fig. 16, the AFM height images show a root-mean-square (RMS) roughness of 1.49 nm for the PTB7-Th:BTPSV-4F film and 2.51 nm for the PTB7-Th:BTPSeV-4F films. The BTPSeV-4F based blend film shows a larger RMS value and better-organized and more-obvious fibrils like morphology, which should result from the strong crystalline property of BTPSeV-4F. The obviously fibrillary networks of the BTPSeV-4F based blend film could be beneficial for charge transport and achieving higher FF. Same phenomenon could also be observed in TEM images in Supplementary Fig. 17. BTPSeV-4F-based blend film formed more obvious phase separation in bi-continuous interpenetrating network than that of BTPSV-4F, which is favorable for charge transport.

### Photovoltaic performance of TOSCs

Obviously, BTPSeV-4F exhibits superiority in both broad absorption (low *E*_g_^opt^) and high photovoltaic performance, which would be a promising acceptor for constructing the rear cell in high performance TOSCs. In addition, the OSC based on PM6:O1-Br is promising for the application as front cell in the TOSCs, and the fabrication conditions of the OSCs were optimized and the results are listed in Supplementary Table 8. Supplementary Fig. 18a shows the *J*–*V* curve of the OSC based on PM6:O1-Br, and the single junction OSC demonstrated a PCE of 15.5% with a *V*_oc_ of 1.04 V, a high FF of 0.744 and an *J*_sc_ of 20.0 mA cm^−2^ (see Table 2). Supplementary Fig. 18b displays the EQE spectrum of the PM6:O1-Br based OSC which shows a high EQE response in the short wavelength range. The integrated *J*_sc_ value from the EQE spectrum is 19.7 mA cm^−2^, which is close to the *J*_sc_ value obtained from its *J*–*V* curve. Obviously, the PM6:O1-Br blend is an efficient front cell active layer with ideal *E*_g_^opt^, reduced *E*_loss_ and high FF simultaneously. According to the method mentioned above, we also studied the detailed energy losses of the PM6:O1-Br based devices, as shown in Supplementary Fig. 19. The *E*_g_^opt^ of PM6:O1-Br based device was calculated to be 1.58 eV, corresponding the Δ*E*_1_ of 0.28 eV. The Δ*E*_2_ values of the OSCs based on PM6:O1-Br were determined to be 0.03 eV by FTPS-EQE (Supplementary Fig. 19a). As shown in Supplementary Fig. 19b, PM6:O1-Br exhibits a high EQE_EL_ of 1.1 × 10^−4^. The corresponding Δ*E*_3_ for the PM6:O1-Br based device is 0.24 eV. So, the total *E*_loss_ of 0.55 eV in medium bandgap system of PM6:O1-Br is also comparable to those of high-performance single-junction devices based on narrower bandgap A-DA’D-A acceptors.

**Table 2:** Photovoltaic parameters of the optimal single junction OSCs and the TOSC, under the illumination of AM 1.5G, 100 mW cm^−2^

Device	*V*_*oc*_ [V]	*J*_*sc*_ [mA·cm^−^^2^]	FF (%)	PCE_max_ (%)
Front cell^a^	1.04 (1.04 ± 0.01)	20.0 (19.7 ± 0.3)	74.4 (74.2 ± 0.3)	15.5 (15.2 ± 0.2)^c^
Rear cell^b^	0.66 (0.66 ± 0.01)	30.1 (28.9 ± 0.6)	71.0 (71.0 ± 0.4)	14.2 (13.9 ± 0.2)
Tandem device	1.69 (1.69 ± 0.01)	15.0 (14.7 ± 0.2)	74.8 (74.6 ± 0.3)	19.0 (18.6 ± 0.3)

^a^The thickness of active layer for the front cell is 100 nm.

^b^The thickness of active layer for the rear cell is 100 nm.

^c^The average values were extracted from 10 devices.

**Fig. 4:**
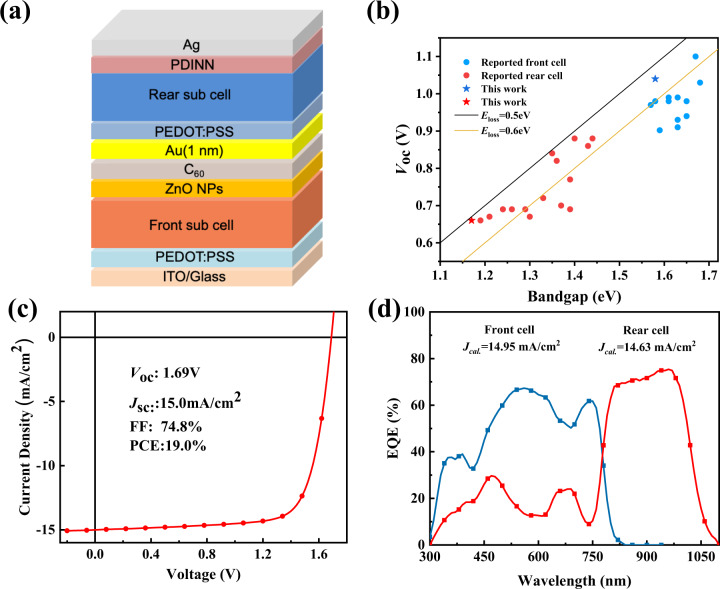
Device structure and photovoltaic performance of the TOSCs. **a** Device architecture of the TOSCs. **b** Plots of *V*_*oc*_ of the OSCs vs *E*_g_^opt^ of the acceptors used in the front cell and rear cell of the TOSCs reported in literatures. **c** *J*–*V* curves of the optimal TOSC under the illumination of AM 1.5G, 100 mW cm^−2^. Source data are provided as a Source Data file. **d** EQE spectra of the front and rear cells of the TOSC. Source data are provided as a Source Data file.

By combining the high *V*_oc_ of the PM6:O1-Br WBG front cell and the broadened absorption of the PTB7-Th:BTPSeV-4F NBG rear cell, the monolithic tandem OSCs were constructed with the device structure of ITO/PEDOT:PSS/Front cell/ZnO NPs/C_60_/Au/PEDOT:PSS/Rear cell/PDINN/Ag (see Fig. 4a). Figure 4b compares the energy loss of the reported rear cell and front cell in the literatures (the corresponding photovoltaic parameters are listed in Supplementary Table 9). It can be seen that the BTPSeV-4F based device shows the narrowest *E*_g_^opt^ and lowest energy loss among all the reported works related to the ultra-narrow bandgap rear cell used in TOSCs. The device with the O1-Br acceptor also exhibits the reduced energy loss, which represents one of the lowest values among the medium bandgap front cells. Obviously, the BTPSeV-4F and O1-Br based devices exhibit great superiority in both *E*_g_^opt^ and energy loss, which would be promising acceptors for constructing the sub-cells in high-performance TOSCs. The best TOSC demonstrated an impressive high PCE of 19.0% with a *V*_*oc*_ of 1.69 V, a *J*_*sc*_ of 15.0 mA cm^−2^ and FF of 74.8%, as shown in Fig. 4c and Table 2. Figure 4d shows the EQE responses of the front and rear cell in the TOSC. The integrated *J*_sc_ values for the front cell and rear cell were 14.95 mA cm^−2^ and 14.63 mA cm^−2^, respectively, which agree well with the *J*_sc_ value measured from the *J*–*V* curve. The high and well-balanced *J*_sc_ can be ascribed to the complementary absorption range and the carefully tuned thickness of each active layer (listed in Supplementary Table 10). However, according to the previous semi-empirical model analysis, PCEs > 25% are predicted for TOSCs with two junctions. So, there is still a lot of room for improving the PCE of TOSCs. First, the FF of narrow and middle bandgap sub cells are obviously lower than high performance single junction OSCs with over 80% FF, which further limit the TOSCs to obtain higher FF. So, it is also a critical issue to develop narrow and medium bandgap systems with ideal molecular packing and morphology in order to get high FF. Second, the ICL with thin metal layer and PEDOT:PSS would result in a large optical loss, which limits the *J*_sc_ of the TOSCs. Thus, the design of a highly optical transparent ICL to maintain efficient recombination while minimizing optical losses remains a major challenge in fabricating high performance TOSCs.

## Discussion

Inspired by recent advances of the A-DA’D-A acceptors for single junction OSC, we developed the wide bandgap acceptor O1-Br and ultra-narrow bandgap acceptor BTPSeV-4F by molecular structure modification of the representative A-DA’D-A type SMAs Y6 and BTPSV-4F respectively, for constructing efficient TOSCs. We designed and synthesized an ultra-narrow bandgap SMA BTPSeV-4F through replacement of terminal thiophene by selenophene in the central fused ring of BTPSV-4F. Compared to original BTPSV-4F, the substitution of selenophene can effectively red-shift the absorption of the BTPSeV-4F film to 1061 nm with a narrow *E*_g_^opt^ of 1.17 eV. The binary OSCs with BTPSeV-4F as acceptor and PTB7-Th as donor demonstrated a higher PCE of 14.2% with a highest *J*_sc_ of 30.1 mA cm^−2^. Importantly, the BTPSeV-4F-based device shows a low energy loss of 0.55 eV for such a low bandgap acceptor. The lower energy loss is attributed to the largely suppressed triplet excitons formation in the PTB7-Th:BTPSeV-4F blend and the low energetic disorder. In the meanwhile, the medium bandgap SMA O1-Br is developed via reducing the intermolecular charge transfer (ICT) effect of Y6. The single junction OSC based on PM6:O1-Br showed a PCE of 15.5% with a high *V*_oc_ of 1.04 V. With adopting BTPSeV-4F and O1-Br as the rear cell and front cell acceptors respectively, the resulting TOSCs demonstrated a high PCE of 19%. All the results indicate that the formation of triplet excitons can be suppressed in the near-infrared-absorbing systems, and the bandgap engineering of A-DA’D-A acceptors is an effective strategy to minimize energy losses in TOSCs.

## Methods

### Materials

The polymer donor PM6 was purchased from Solarmer Materials Inc. The polymer donor PTB7-Th was purchased from 1-Material Inc. BTPSe-CHO, BTPSV-4F and O1-CHO were prepared according to the literatures^37,38,40^. IC-Br and IC-2F were purchased from Derthon Optoelectronic Materials Co., Ltd. Other chemicals and solvents were obtained from J&K, Energy Chemical, and Sigma Aldrich Chemical Co., respectively. All of the reagents and commercial compounds were used as received. The NMR spectra of acceptors are shown in Supplementary Figs. 20–25. The synthetic routes of acceptor BTPSeV-4F and O1-Br are shown in Supplementary Figs. 26 and  27, respectively, and the detailed synthesis processes are described in the following.

### Synthesis of BTPSeV-CHO

To a solution of the mixture of BTPSe-CHO (219 mg, 0.2 mmol) and tributyl(1,3-dioxolan-2-ylmethyl)phosphonium bromide (94 mg, 0.22 mmol) in anhydrous tetrahydrofuran was added sodium hydride (60% dispersed in mineral oil, 0.6 mmol) under an argon gas atmosphere, and the resulting turbid solution was stirred at room temperature for 16 h. After completion of the reaction, the reaction was quenched using a 10% HCl solution under cooling and the reaction mixture was brought to acidic pH and stirred at room temperature for 4–5 h. The contents of the reaction flask were concentrated, and the organic contents were extracted into ethyl acetate. The organic layer was washed with water followed by brine, dried with anhydrous MgSO_4_, filtered, and evaporated to dryness to afford the crude aldehyde. Then after purification by silica gel column chromatography using dichloromethane gave the product as a red solid (187 mg, 79% yield). ^1^H-NMR (400 MHz, Chloroform-*d*) δ (p.p.m.) 9.71 (d, *J* = 7.6 Hz, 2H), 7.81 (d, *J* = 15.0 Hz, 2H), 6.40 (dd, *J* = 15.0, 7.6 Hz, 2H), 4.55 (d, *J* = 7.8 Hz, 4H), 2.99 (t, *J* = 7.8 Hz, 4H), 2.06 (s, 2H), 1.85 (t, *J* = 7.7 Hz, 4H), 1.48–1.18 (m, 34H), 1.1-0.71 (m, 34H), 0.70–0.65 (m, 16H).^13^C-NMR (101 MHz, Chloroform-*d*) δ (p.p.m.) 191.30, 146.48, 144.42, 144.06, 143.43, 143.20, 139.30, 139.28, 135.55, 132.07, 132.05, 125.31, 124.39, 124.35, 122.33, 110.97, 76.32, 76.00, 75.68, 54.05, 37.94, 33.53, 30.89, 30.54, 29.39, 29.25, 29.15, 29.07, 28.75, 28.64, 28.59, 28.54, 28.42, 28.31, 27.04, 26.81, 24.28, 24.03, 21.73, 21.67, 21.44, 21.41, 13.09, 12.93, 12.73, 12.68.

### Synthesis of BTPSeV-4F

BTPSeV-CHO (178 mg, 0.15 mmol), 2-(5, 6-difluoro-3-oxo-2,3-dihydro-1H-inden-1- ylidene) malononitrile (116 mg, 0.50 mmol), pyridine (0.5 mL) and chloroform (20 mL) were dissolved in a round bottom flask under nitrogen. The mixture was stirred at room temperature overnight. Then, the mixture was purified with column chromatography using dichloromethane to give BTPSeV-4F (192.6 mg, 75% yield). ^1^H-NMR (400 MHz, CDCl_3_): δ (p.p.m.) 8.60–8.41 (m, 6H), 7.76 (dd, *J* = 11.0, 2.3 Hz, 2H), 7.67 (t, *J* = 7.5 Hz, 2H), 4.59 (d, *J* = 7.9 Hz, 4H), 3.02 (t, *J* = 7.8 Hz, 4H), 2.21–2.00 (m, 2H), 1.85 (p, *J* = 7.6 Hz, 4H), 1.54–1.21 (m, 32H), 1.20–0.35 (m, 52H). ^13^C-NMR (101 MHz, Chloroform-*d*): δ (p.p.m.) 187.25, 147.60, 147.51, 146.70, 145.96, 141.24, 140.24, 133.43, 127.47, 126.24, 123.31, 114.85, 114.40, 114.37, 112.93, 69.42, 55.39, 38.94, 31.92, 31.55, 30.57, 30.43, 30.32, 30.21, 29.97, 29.82, 29.66, 29.63, 29.56, 29.48, 29.36, 29.34, 28.02, 27.89, 25.39, 25.24, 22.77, 22.73, 22.69, 22.48, 14.11, 13.98, 13.76, 13.73. HRMS (TOF) *m*/*z* calcd. for [M]^+^ C_86_H_90_F_4_N_8_O_2_S_3_Se_2_ 1711.58, found 1711.5804.

### Synthesis of O1-Br

O1-CHO (128.4 mg, 0.11 mmol) and 2-(5-bromo-3-oxo-2,3-dihydro-1H-inden-1-ylidene) malononitrile (150 mg, 0.65 mmol) were dissolved in CHCl_3_ (25 mL) under a nitrogen atmosphere. 0.6 mL pyridine was added and refluxed for 12 h. Then the mixture was purified using column chromatography on silica gel employing petroleum ether/CHCl_3_ (1:1) as an eluent, yielding a dark blue solid O1-Br (137 mg, 73% yield). ^1^H-NMR (400 MHz, Chloroform-*d*) δ 9.35 (s, 2H), 8.50 (d, *J* = 8.4 Hz, 2H), 7.97 (d, *J* = 1.9 Hz, 2H), 7.79 (dd, *J* = 8.4, 2.0 Hz, 2H), 4.78 (q, *J* = 7.5, 7.1 Hz, 8H), 2.21–1.97 (m, 6H), 1.65–1.51 (m, 5H), 1.46–1.26 (m, 31H), 1.18–0.83 (m, 35H), 0.75–0.70 (m, 13H). ^13^C-NMR (101 MHz, CDCl_3_) δ 187.36, 162.30, 159.75, 147.40, 138.69, 138.26, 137.59, 137.15, 134.68, 134.40, 131.75, 128.81, 128.22, 126.40, 126.19, 120.21, 117.95, 115.42, 114.96, 113.06, 74.17, 67.24, 55.81, 39.34, 31.95, 31.61, 31.58, 30.53, 30.44, 29.71, 29.69, 29.63, 29.56, 29.48, 29.45, 29.38, 29.25, 28.02, 27.91, 25.82, 25.51, 25.38, 22.83, 22.80, 22.71, 22.53, 22.52, 14.12, 14.05, 14.04, 13.78, 13.76. HRMS (TOF) *m*/*z* calcd. for [M]^+^ C_92_H_106_N_8_O_2_S_5_Br_2_ 1709.63, found 1709.54655.

### Material characterization

The NMR spectra were measured using Bruker AVANCE 400 MHz spectrometer. Electrochemical measurements were performed under nitrogen in a solution of tetra-*n*-butylammonium hexafluorophosphate ([*n-*Bu_4_N]^+^[PF_6_]^−^) (0.1 M) in CH_3_CN employing a computer-controlled CHI660C electrochemical workstation, with glassy carbon working electrode coated with sample films, an Ag/AgCl reference electrode, and a platinum-wire auxiliary electrode. The redox potential of a ferrocenium/ferrocene (FeCp^2+/0^) couple was used as an internal standard, the sample molecules are not soluble in acetonitrile. The UV-vis absorption spectra were measured on a Hitachi U-3010 UV–vis spectrophotometer. For the measurement of films, the acceptors and blend films were prepared by spin-coating the corresponding solutions on quartz plates.

### Device fabrication of single junction OSCs

The conventional structure OSCs based on PTB7-Th:BTPSV-4F and PTB7-Th:BTPSeV-4F were fabricated with device architecture of ITO/PEDOT:PSS/Active Layer/PDINN/Ag. Prior to fabrication, the ITO substrates were cleaned using detergent, deionized water, acetone and isopropanol consecutively for 15 min in each step, and then treated in an ultraviolet ozone generator for 15 min before being spin coating PEDOT:PSS (Baytron P AI4083) at 4500 rpm with a layer of 20 nm thick. After baking the PEDOT:PSS layer in air at 150 °C for 20 min, the substrates were transferred to a glovebox filled with nitrogen, in which the active layer of PTB7-Th:BTPSeV-4F (or PTB7-Th:BTPSV-4F) (1:1.6 w/w) (16 mg/mL in total, from CF solution with 0.5% CN solvent additive) is spin-coated onto the PEDOT:PSS layer at 3000 rpm. The thickness of the active layer is ca. 100 nm. Then the active layer is annealed at 100 °C for 5 min for the devices with thermal annealing treatment. After cooled down, 30 µL 1 mg/mL PDINN methanol solution was spin-coated onto the organic active layers at spin speed of 3000 r.p.m. for 30 s. Finally, 100 nm Ag electrode was evaporated under 1 × 10^−6^ mbar vacuum. For the front cell, PM6:O1-Br (1:1, w/w) (16 mg/mL in total, from CF solution with 0.6% CN solvent additive) was spin coated onto PEDOT:PSS at 3300 rpm. The thickness of the active layer is ca. 100 nm. After that, the active layer is annealed at 100 °C for 5 min for the devices with thermal annealing treatment. After cooled down, 30 µL 1 mg/mL PDINN methanol solution was spin-coated onto the organic active layers at spin speed of 3000 r.p.m. for 30 s. Finally, 100 nm Ag electrode was evaporated under 1 × 10^−6^ mbar vacuum.

### Device fabrication of tandem OSCs

The tandem OSCs were fabricated with an architecture of ITO/ PEDOT:PSS/PM6:O1-Br/ZnO NPs/C_60_/Au/PEDOT:PSS /PTB7-Th:BTPSeV-4F /PDINN/Ag. The PM6:O1-Br active layers were fabricated via the same process as the single-junction OSCs with different thicknesses. Then, ZnO nanoparticles (10 nm) in IPA solution was spin-coated and annealed at 120 °C for 5 min in the glovebox. Then, 5 nm C_60_ and 1 nm Au were sequentially evaporated under 1 × 10^−4^ Pa vacuum. The PEDOT:PSS solution was then spin-casted (5000 rpm) and annealed at 130 °C for 2 min. Then, the PTB7-Th:BTPSeV-4F active layers were fabricated via the same process as the single junction devices. 30 µL 1 mg/mL PDINN methanol solution was spin-coated onto the organic active layers at spin speed of 3000 r.p.m. for 30 s. Finally, 100 nm Ag electrode was evaporated under 1 × 10^−6^ mbar vacuum. The device area of the OSCs was 6.0 mm^2^, which was defined by optical microscope (Olympus BX51). In order to accurately measure the photocurrent, mask with an area of 4.80 mm^2^ was used to define the effective area of the OSCs.

### Device characterization

The current density–voltage (*J*–*V*) characteristics of the OSCs were measured in a nitrogen glove box with a Keithley 2450 Source Measure unit. Class AAA Solar Simulator (SS-X100R, Enlitech) with a 450 W xenon lamp and an air mass (AM) 1.5 filter was used as the light source. The light intensity was calibrated to 100 mW cm^−2^ by a SRC−2020 reference cell with a KG2 filter window. The mismatch factor is calculated to be 1.007 for the tandem devices. The input photon to converted current efficiency (IPCE) was measured by Solar Cell Spectral Response Measurement System QE-R3-011 (Enli Technology Co., Ltd., Taiwan). The light intensity at each wavelength was calibrated with a standard single-crystal Si photovoltaic cell. To measure the rear and front cell, light bias obtained by 550 nm short wave pass filters and 850 nm long wave pass filters were selected to excite (saturate) the front and rear cells, respectively.

### Charge transport characterization

In the photo-CELIV measurement, the delay time is set to 0 s, the light intensity is 100%, the light-pulse length is 100 µs, finally the sweep ramp rate rises from 20 V/ms to 100 V/ms. The charge carrier mobilities were measured with the device structure of ITO/PEDOT:PSS/active layer/MoO_3_/Ag for hole-mobility and ITO/ZnO/active layer/PDINO/Al for electron-mobility devices. The hole and electron mobilities are calculated according to the space charge limited current (SCLC) method equation:

4 $$J=9\mu {\varepsilon }_{{{{{{\rm{r}}}}}}}{\varepsilon }_{0}{V}^{2}/8{d}^{3}$$

where *J* is the current density, *µ* is the hole or electron mobility, *V* is the internal voltage in the device, *ε*_r_ is the relative dielectric constant of active layer material, *ε*_0_ is the permittivity of empty space, and *d* is the thickness of the active layer.

### Energy loss characterization

A modified Bruker Vertex 70 is used for FTPS EQE spectra. The device was connected with the current amplifier (SR570) to amplify the signal from the device. The data is calibrated with the standard Si and Ge detector. SR303i spectrometer is used to measure the EL. The spectrometer is attached with the newton EMCCD-Si and iDus InGaAs array detector. During the measurement, EMCCD was kept at −40 °C and InGaAs detector kept at −60 °C. The bias of the EL measurement was applied on the devices using a Keithley 2400 Source Meter. The spectrum was calibrated with the standard light source. EQE_EL_ values were obtained from an in-house-built system including a Hamamatsu silicon photodiode 1010B, a Keithley 2400 Source Meter to provide voltage and injected current, and a Keithley 485 Picoammeter to measure the emitted light intensity.

### Transient absorption spectroscopy

To carry out femtosecond transient absorption spectroscopy, the fundamental output from a Yb:KGW laser (1030 nm, 220 fs Gaussian fit, 100 kHz, Light Conversion Ltd) was separated into two light beams. One was introduced into a NOPA (ORPHEUS-N, Light Conversion Ltd) to produce a specific wavelength for the pump beam (here, we used 900 nm), the other was focused onto a YAG plate to generate a white light continuum as the probe beam. The pump and probe overlapped on the sample at a small angle lesser than 10°. The probe light transmitted by the sample was collected by a linear CCD array and a spectrograph (Princeton Instruments) with a liquid-N_2_-cooled CCD (PrLoN-IR) at −100 °C.

### Atomic force microscope (AFM)

The atomic force microscope used is a VistaScope from Molecular Vista, Inc., operated in dynamic mode using commercial gold-coated silicon cantilevers (NCHAu) from Nanosensors using tapping mode.

### Grazing incident wide-angle X-ray scattering (GIWAXS)

Grazing-incidence wide-angle X-ray scattering (GIWAXS) measurements were conducted at Advanced Light Source (ALS), Lawrence Berkeley National Laboratory, Berkeley, CA at the beamline 7.3.3^50^. Data was acqured at the critical angle (0.13^0^) of the film with a hard X-ray energy of 10 keV. X-ray irradiation time was 30–60 s, dependent on the saturation level of the detector. Beam center was calibrated using AgBr powder and the sample-to-detector distance was about 280 mm. The π–π coherence lengths (L) are estimated based on the Scherrer Equation (*L* = 2π*K*/FWHM), where K is the shape factor (here we use 0.9), and FWHM is the full width at half maximum of the (010) diffraction peaks.

### DFT calculation

ORCA (version 4.2.1) was utilized for all the DFT calculations mentioned in this article^60^. The molecular geometries were optimized with a range-separated hybrid functional ωB97X-D3 and a basis set of def2-SVP. The straight and branched alkyl chains were simplified to methyl and isopropyl, respectively, for saving time. The single point energies and gradients were further calculated with a perturbatively corrected double-hybrid functional of PWPB95 with a dispersion correction of D3BJ and a basis set of def2-TZVPP^61,62^.The band gaps were calculated with a functional of B3PW and a basis set of def2-TZVP. The excited states incorporating with SMD solvation model were calculated by TD-DFT with a hybrid functional of B3LYP and a basis set of def2-SVP^63^. The localized orbital locator (LOL), Mayer bond order were analyzed by Multiwfn and VMD (version 1.9.3) for visualization^64^.

### Reporting summary

Further information on research design is available in the Nature Portfolio Reporting Summary linked to this article.

## Supplementary information

Supplementary Information

Solar Cells Reporting Summary

## Data Availability

The data that support the findings of this study are presented in Supplementary Information. The source data underlying Figs. 1c, 2a–f, and 4c, d are provided in the Source Data files with this paper or available from the corresponding author on request. Source data are provided with this paper.

## Source data

Source Data
