# Supplementary material for: Near-infrared absorbing acceptor with suppressed triplet exciton generation enabling high performance tandem organic solar cells
Source: Nat Commun. 2023 Mar 4;14:1236. doi: 10.1038/s41467-023-36917-y (PMC9985646; doi:10.1038/s41467-023-36917-y)
Supplement: Supplementary file 1 — Supplementary Information [file 41467_2023_36917_MOESM1_ESM.pdf]

SUPPLEMENTARY INFORMATION

Near-infrared absorbing acceptor with suppressed triplet exciton generation enabling high performance tandem organic solar cells

SUPPLEMENTARY FIGURES

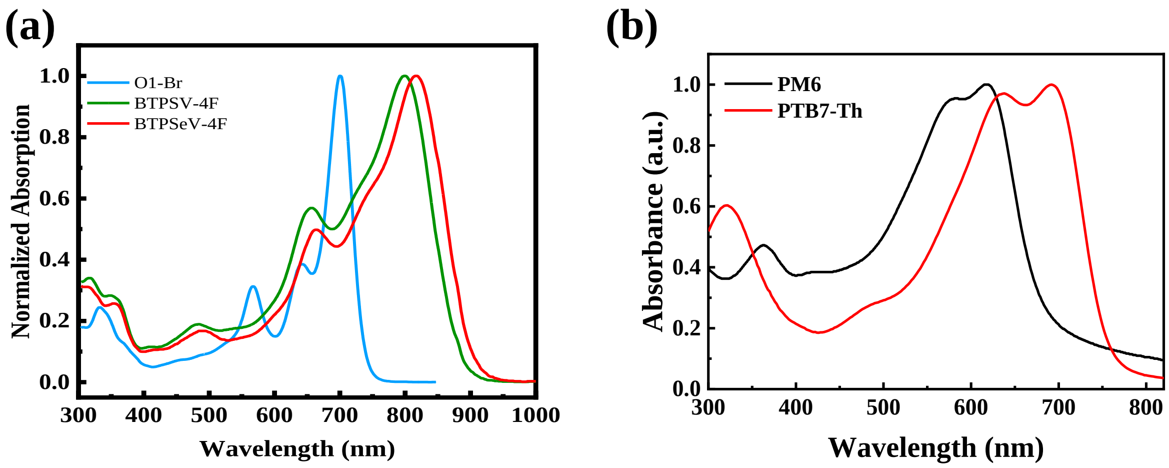

Supplementary Figure 1. **UV-Vis absorption spectra**. (a) Absorption spectra of O1-Br, BTPSV-4F and BTPSeV-4F in chloroform solutions. (b) Absorption spectra of the PM6 and PTB7-Th films.

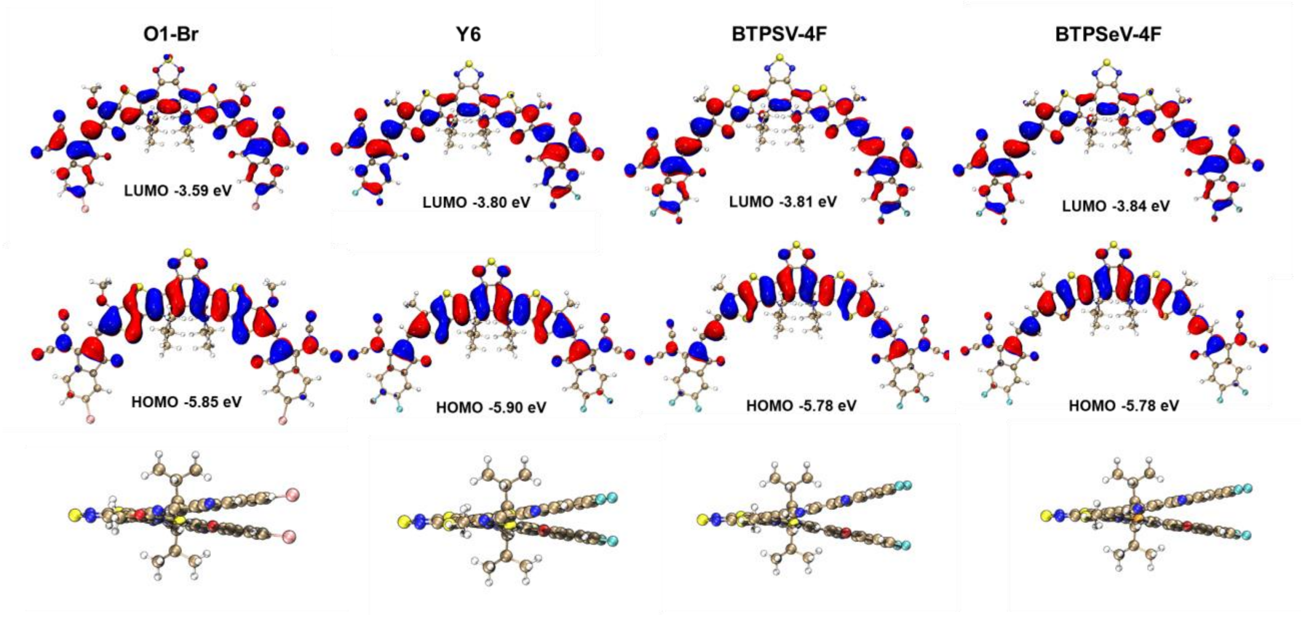

Supplementary Figure 2. Simulated frontier molecular orbitals and optimized geometries obtained by DFT calculations for simplified molecules of O1-Br, Y6, BTPSV-4F and BTPSeV-4F.

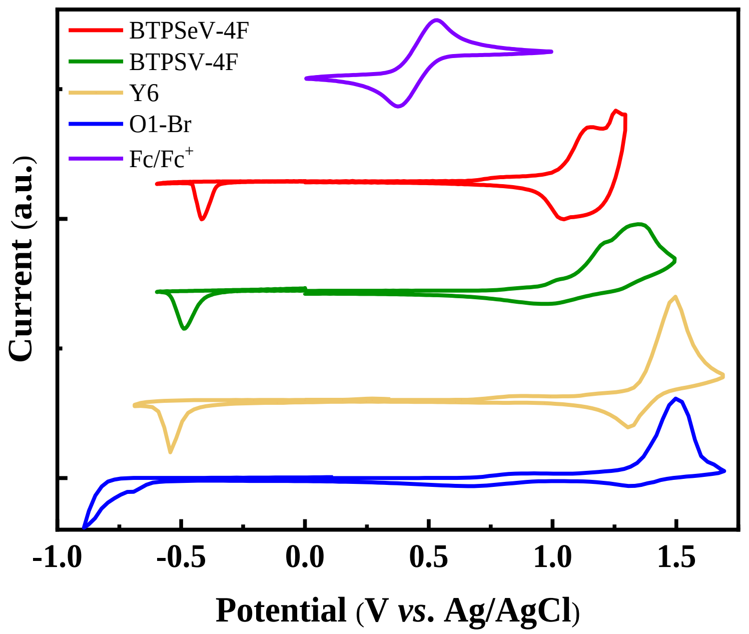

Supplementary Figure 3. Cyclic voltammograms of O1-Br, Y6, BTPSV-4F and BTPSeV-4F films.

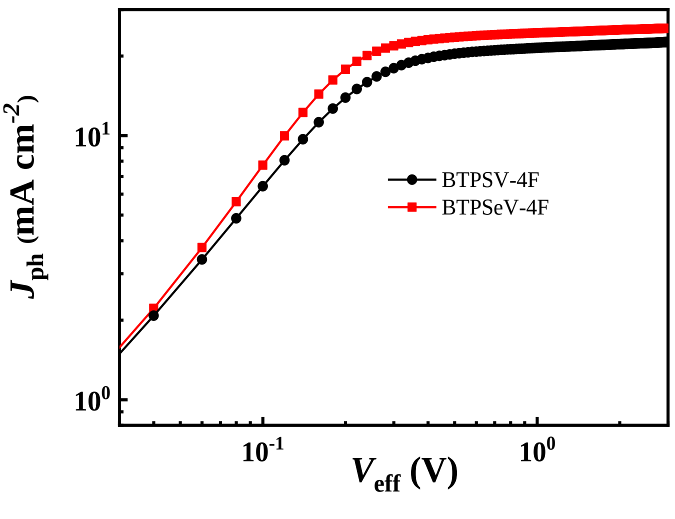

Supplementary Figure 4. J_{ph} versus V_{eff} of the OSCs based on PTB7-Th:BTPSV-4F and PTB7-Th:BTPSV-4F.

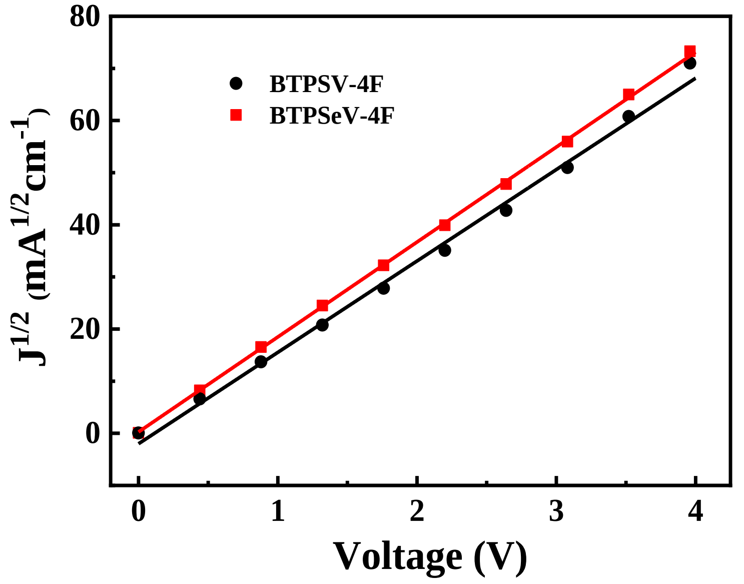

Supplementary Figure 5 Mobilities measurement plots of electron-only devices based on different acceptors.

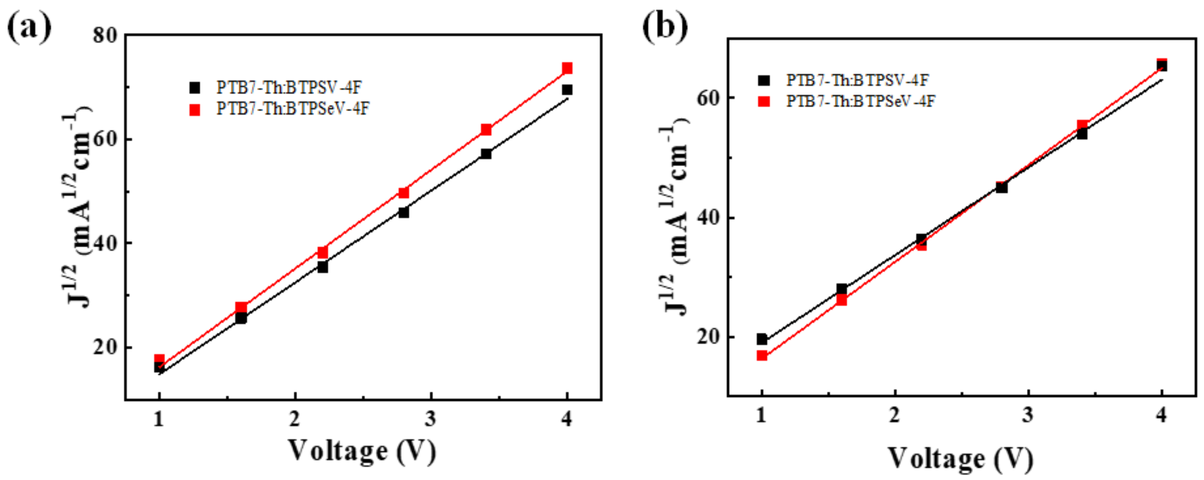

Supplementary Figure 6. **Mobility measurements of the devices.** Mobilities measurement plots of (a) electron-only devices and (b) hole-only devices based on different blend active layers.

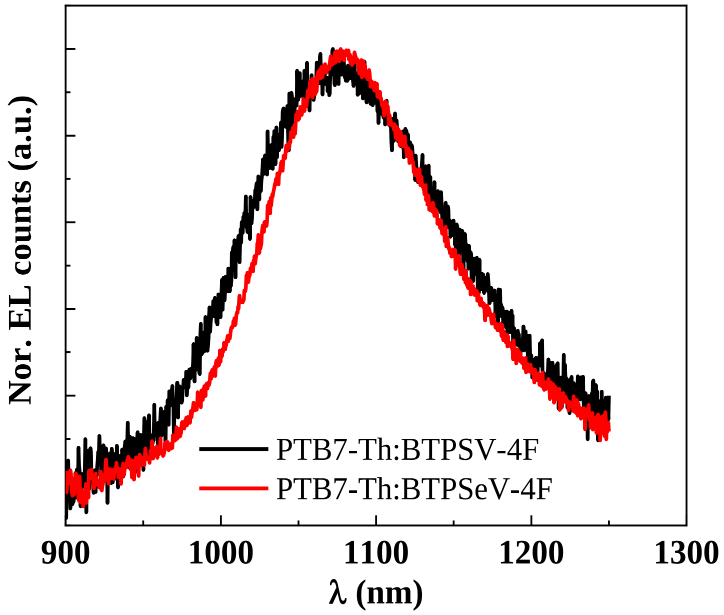

Supplementary Figure 7. Normalized electroluminescence (EL) of the devices based on PTB7-Th:BTPSV-4F and PTB7-Th:BTPSeV-4F.

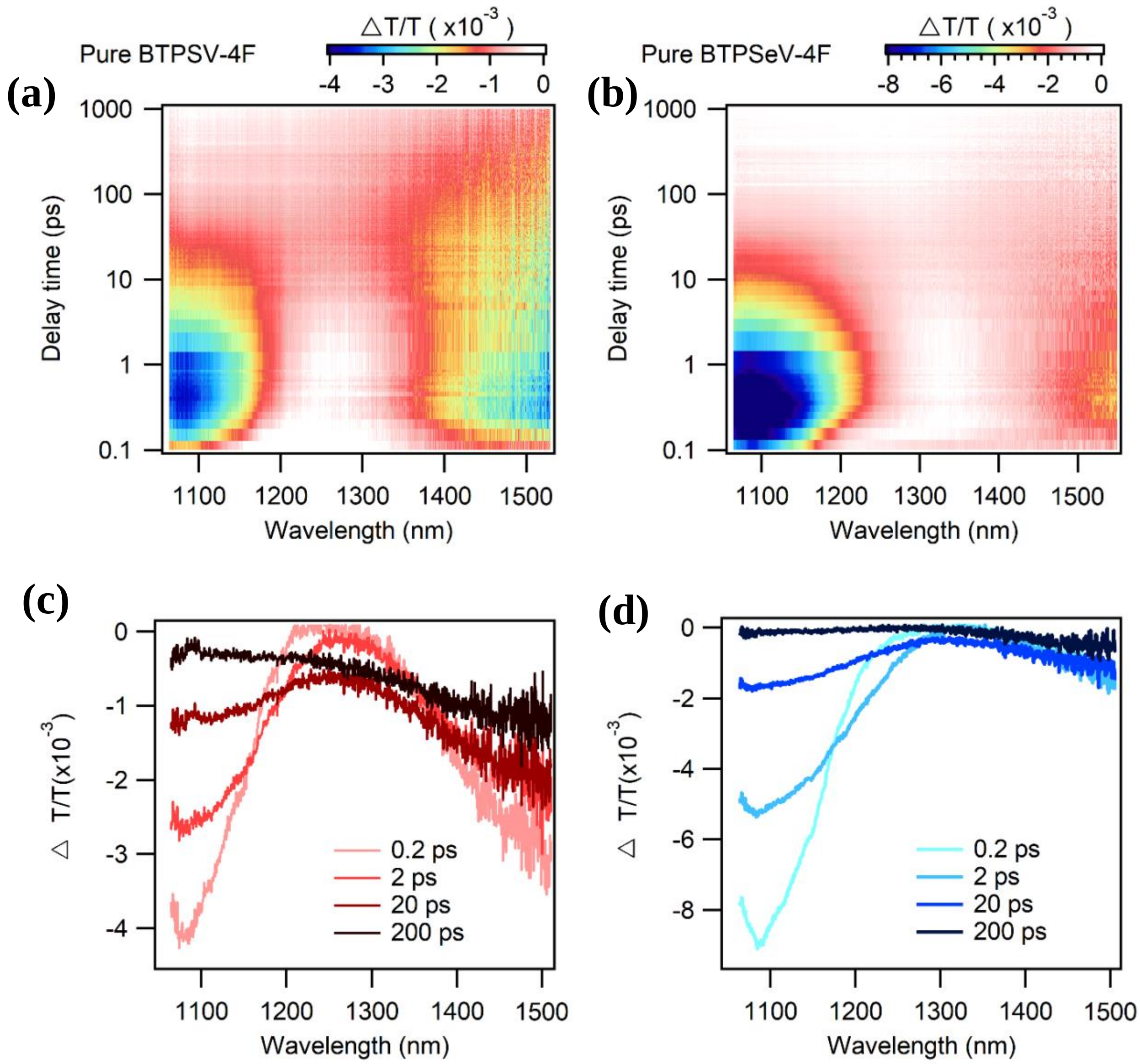

Supplementary Figure 8. **TA spectra of acceptors in NIR region.** (a) TA spectra of pure BTPSV-4F film and (b) pure BTPSeV-4F film under 900 nm excitation at ~10 uJ/cm^2. Representative TA spectra at the indicated delay times for (c) pure BTPSV-4F film and (d) pure BTPSeV-4F film in NIR region.

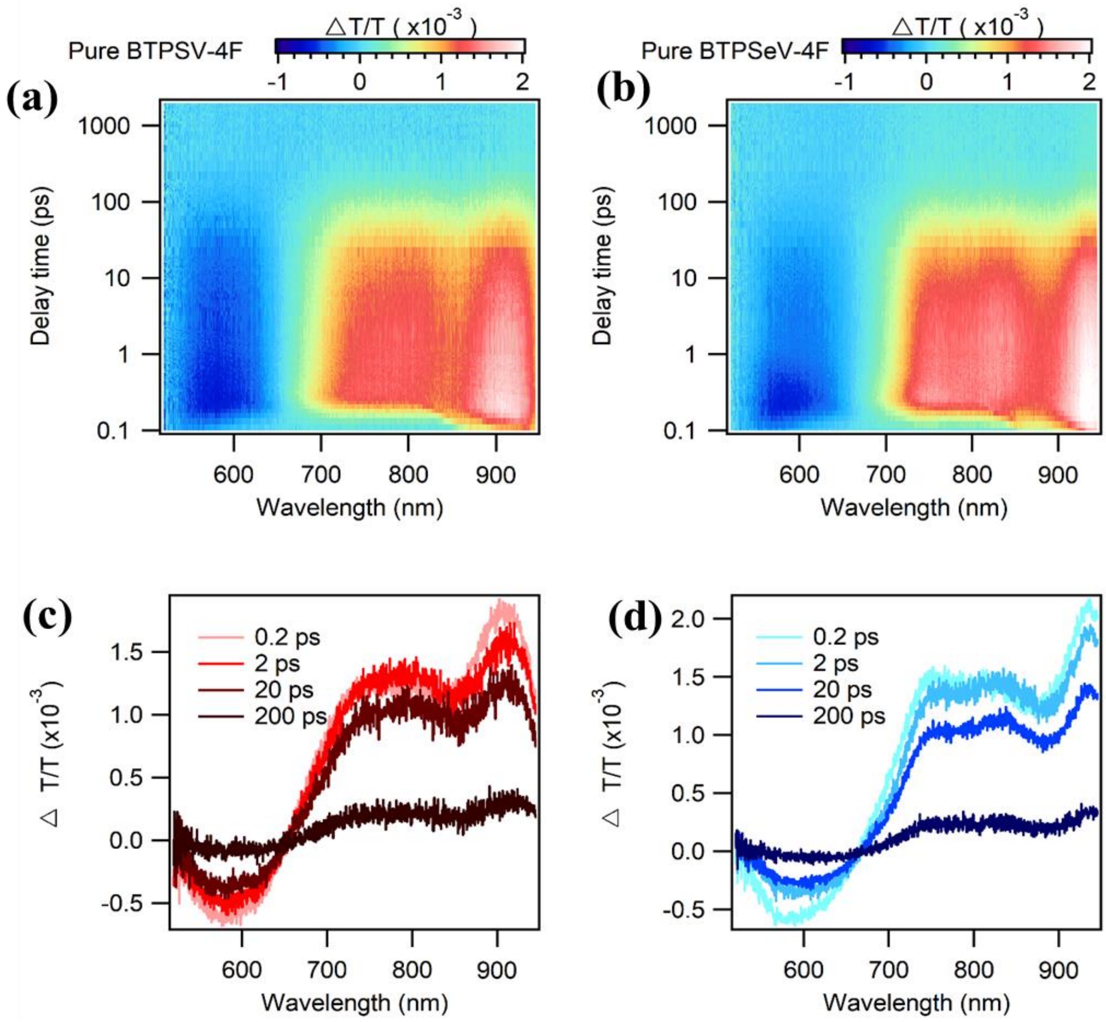

Supplementary Figure 9. **TA spectra of acceptors.** Color plot of TA spectra of (a) pure BTPSV-4F film and (b) pure BTPSeV-4F film under 900 nm excitation at VIS-NIR region. Representative TA spectra at the indicated delay times for (c) pure BTPSV-4F film and (d) pure BTPSeV-4F film.

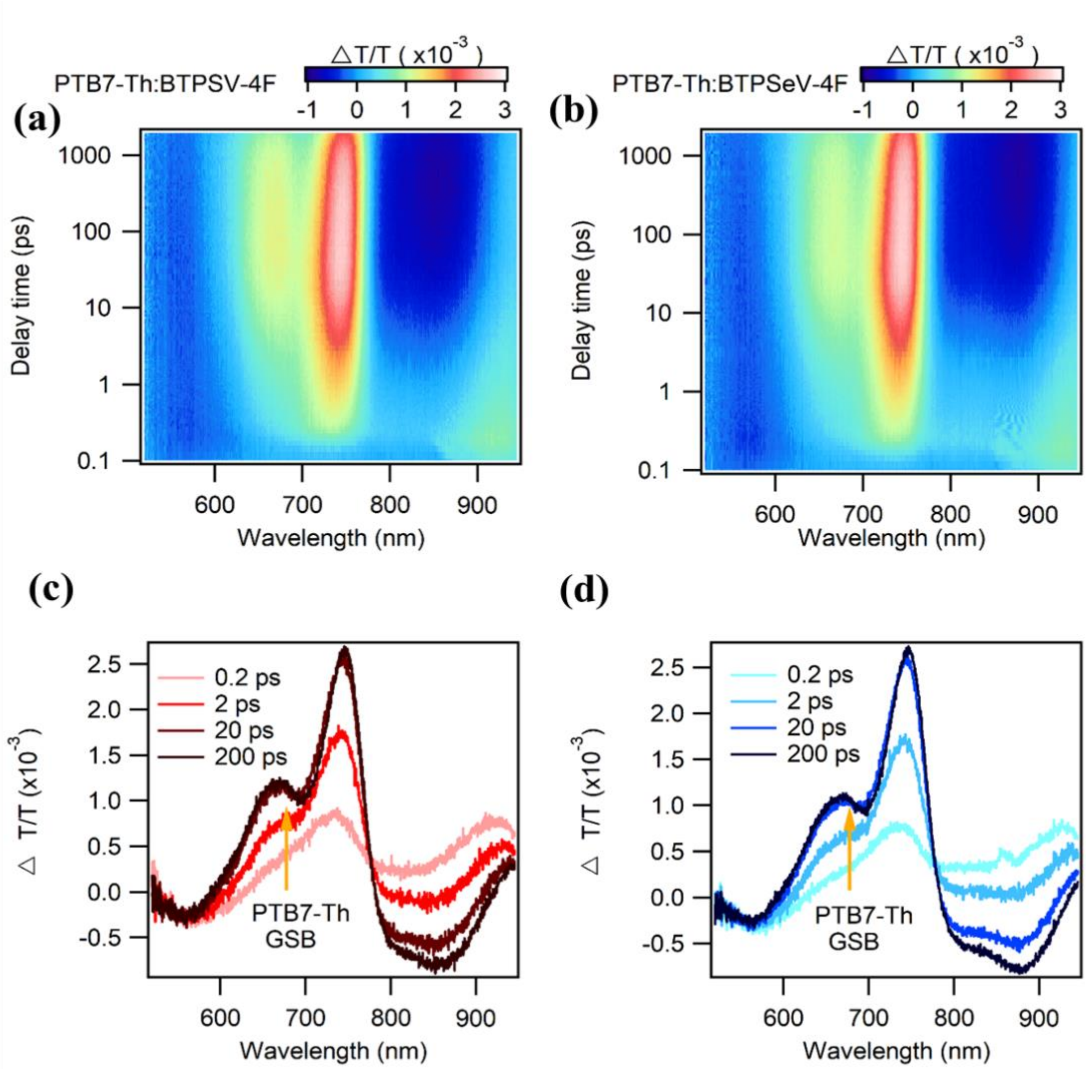

Supplementary Figure 10. **TA spectra of blend films.** Color plot of TA spectra of (a) PTB7-Th:BTPSV-4F blend film and (b) PTB7-Th:BTPSeV-4F blend film under 900 nm excitation at VIS-NIR region. Representative TA spectra at the indicated delay times for (c) PTB7-Th:BTPSV-4F blend film and (d) PTB7-Th:BTPSeV-4F blend film.

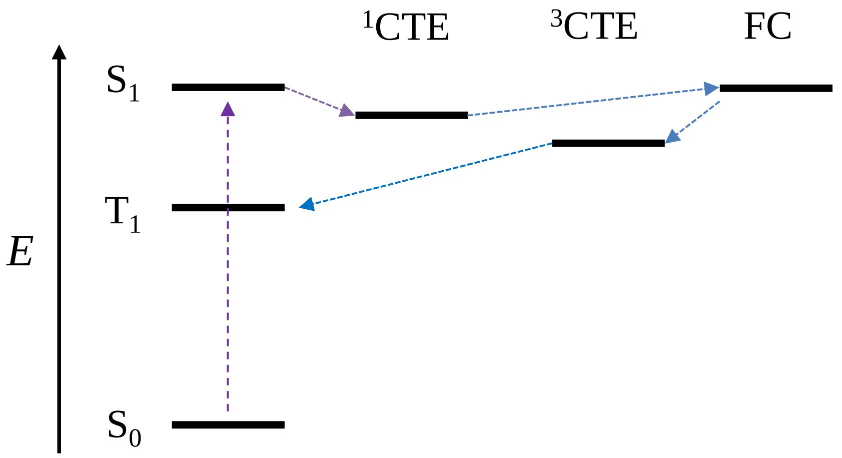

Supplementary Figure 11. Schematic diagram of triplet excited states formation in organic solar cells.[21]

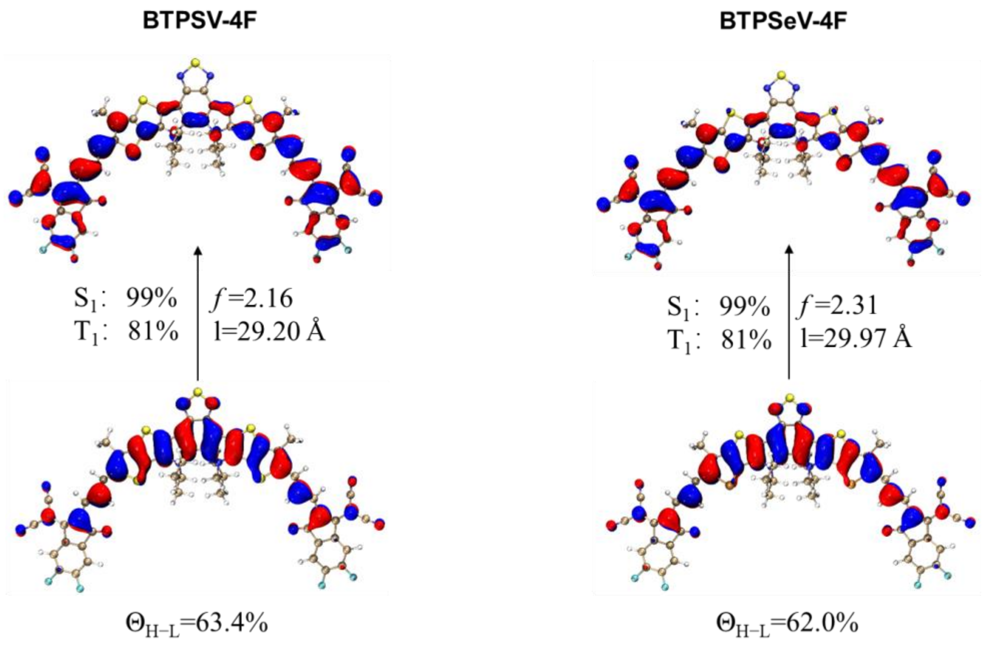

Supplementary Figure 12. Pictorial representation of the HOMO and LUMO wavefunctions of BTPSV-4F and BTPSeV-4F. The weights of the HOMO → LUMO transition in the S_1 and T_1 excitations, oscillator strength and the overlap between HOMO and LUMO ($\Theta_{H\text{–}L}$) are provided.

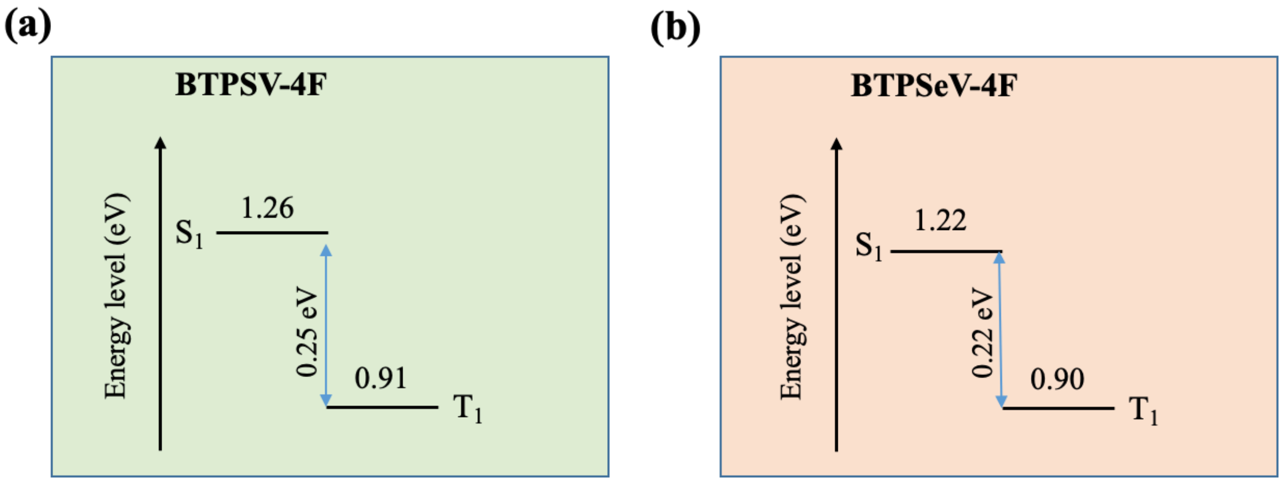

Supplementary Figure 13. **Singlet−triplet energy gap of acceptors.** (a) Vertical excitation energies of BTPSV-4F (b) Vertical excitation energies of BTPSV-4F. Inset: double arrows represent the energy gap between T_1 and S_1 states.

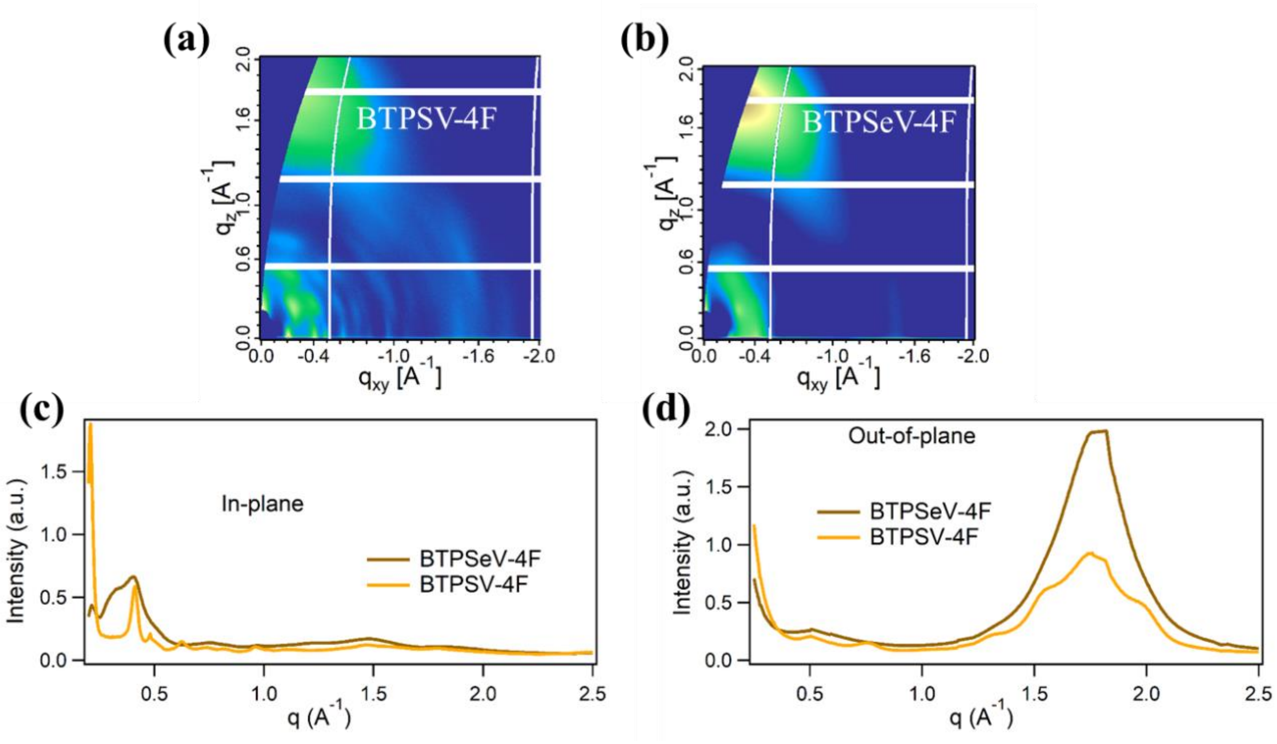

Supplementary Figure 14. **Results of GIWAXS measurements of acceptors.** (a-b) GIWAXS images and (c-d) corresponding line cuts of the GIWAXS images of neat films of BTPSV-4F and BTPSeV-4F.

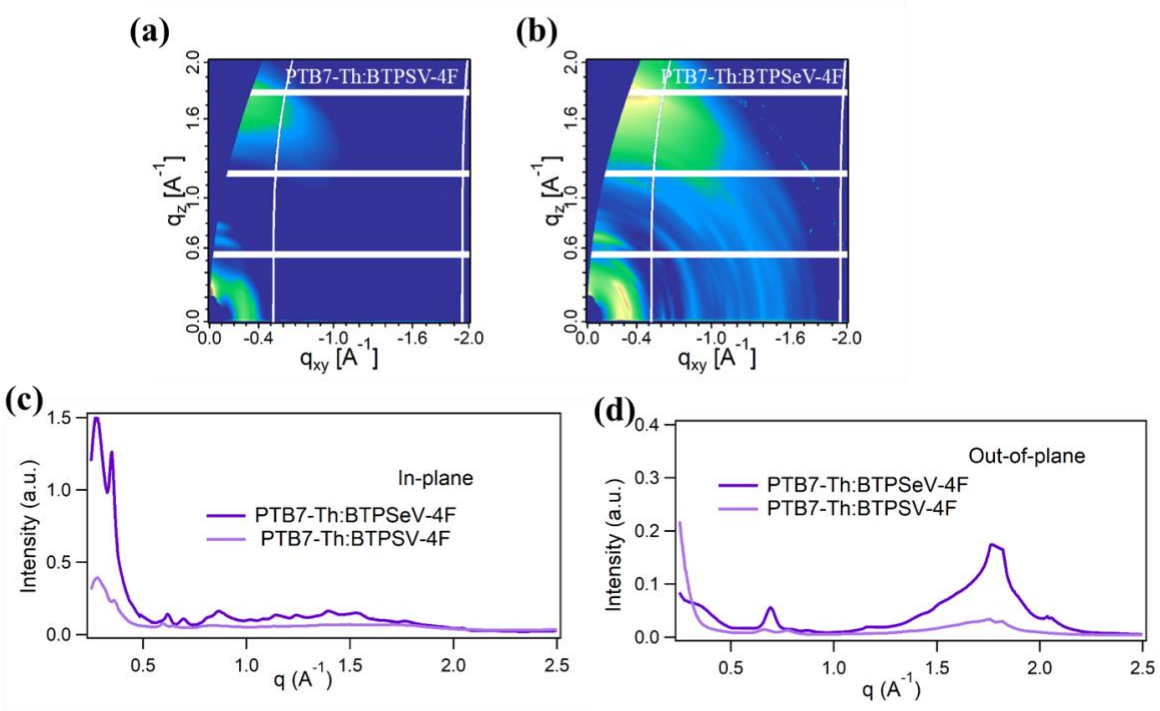

Supplementary Figure 15. **Results of GIWAXS measurements of blend films.** (a-b) GIWAXS images and (c-d) corresponding line cuts of the GIWAXS images of blend films of PTB7-Th:BTPSV-4F and PTB7-Th:BTPSeV-4F.

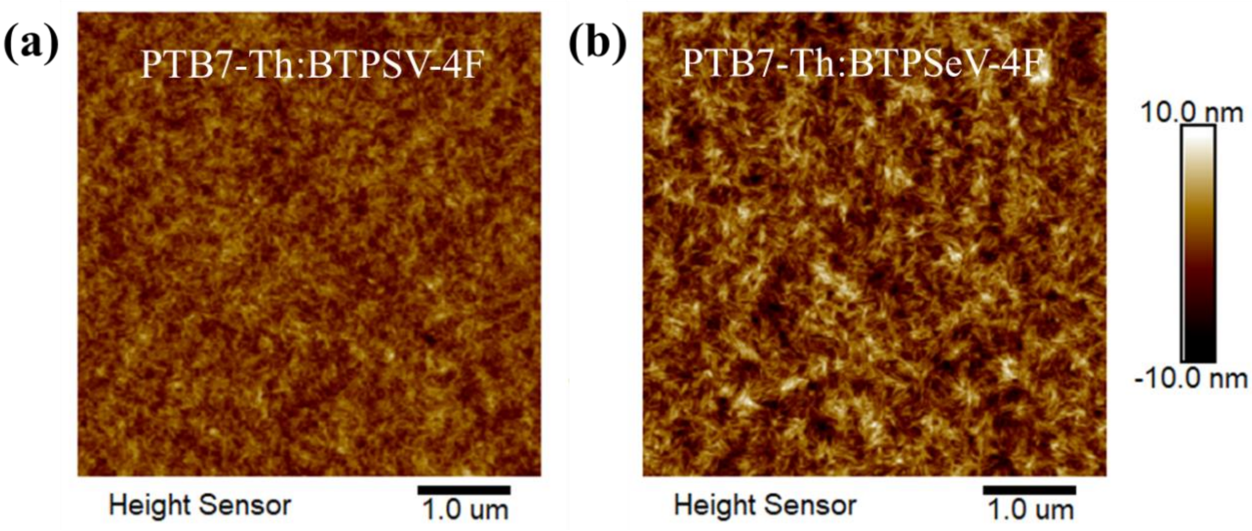

Supplementary Figure 16. AFM height images of (a) PTB7-Th:BTPSV-4F and (b) PTB7-Th:BTPSeV-4F films.

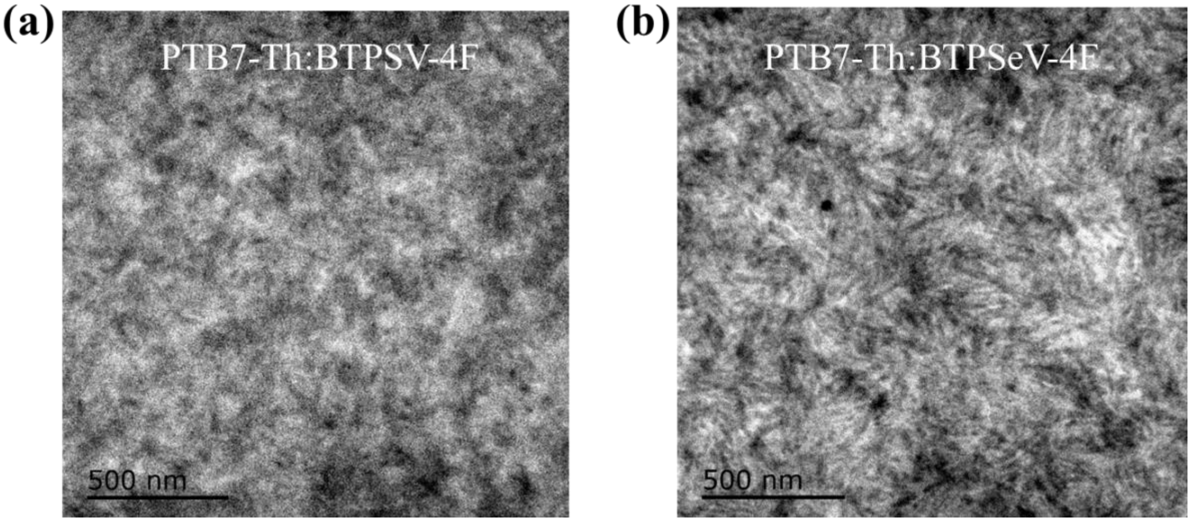

Supplementary Figure 17. TEM images of (a) PTB7-Th:BTPSV-4F and (b) PTB7-Th:BTPSeV-4F films.

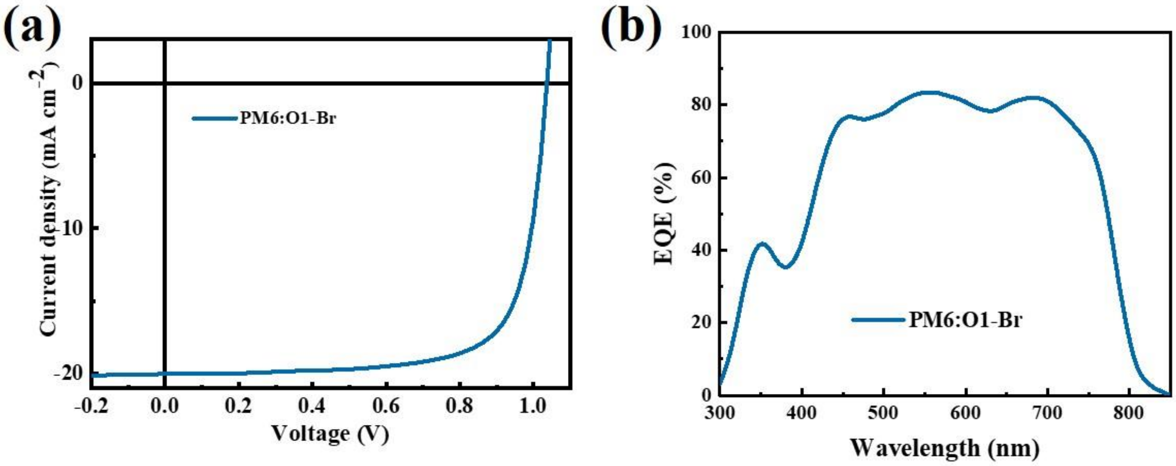

Supplementary Figure 18. **Photovoltaic performance of the front cell.** (a) The *J*-*V* curve of the OSC based on PM6:O1-Br with 100 nm active layer under the illumination of AM1.5G, 100 mW cm^{-2}. (b) EQE spectrum of the corrsponding OSC.

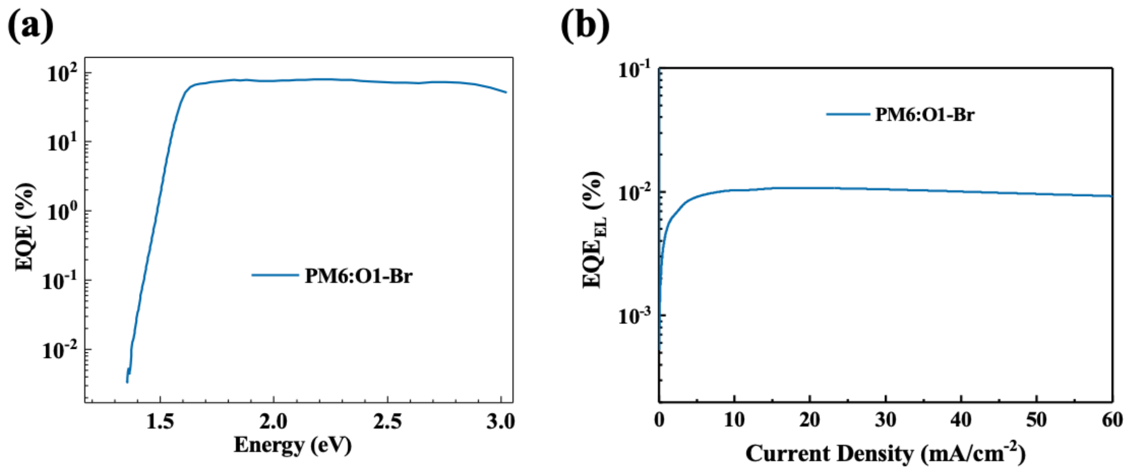

Supplementary Figure 19. **Energy loss of the front cell.** (a) FTPS-EQE curve of the OSC based on PM6:O1-Br. (b) EQE_{EL} spectrum of the OSC based on PM6:O1-Br.

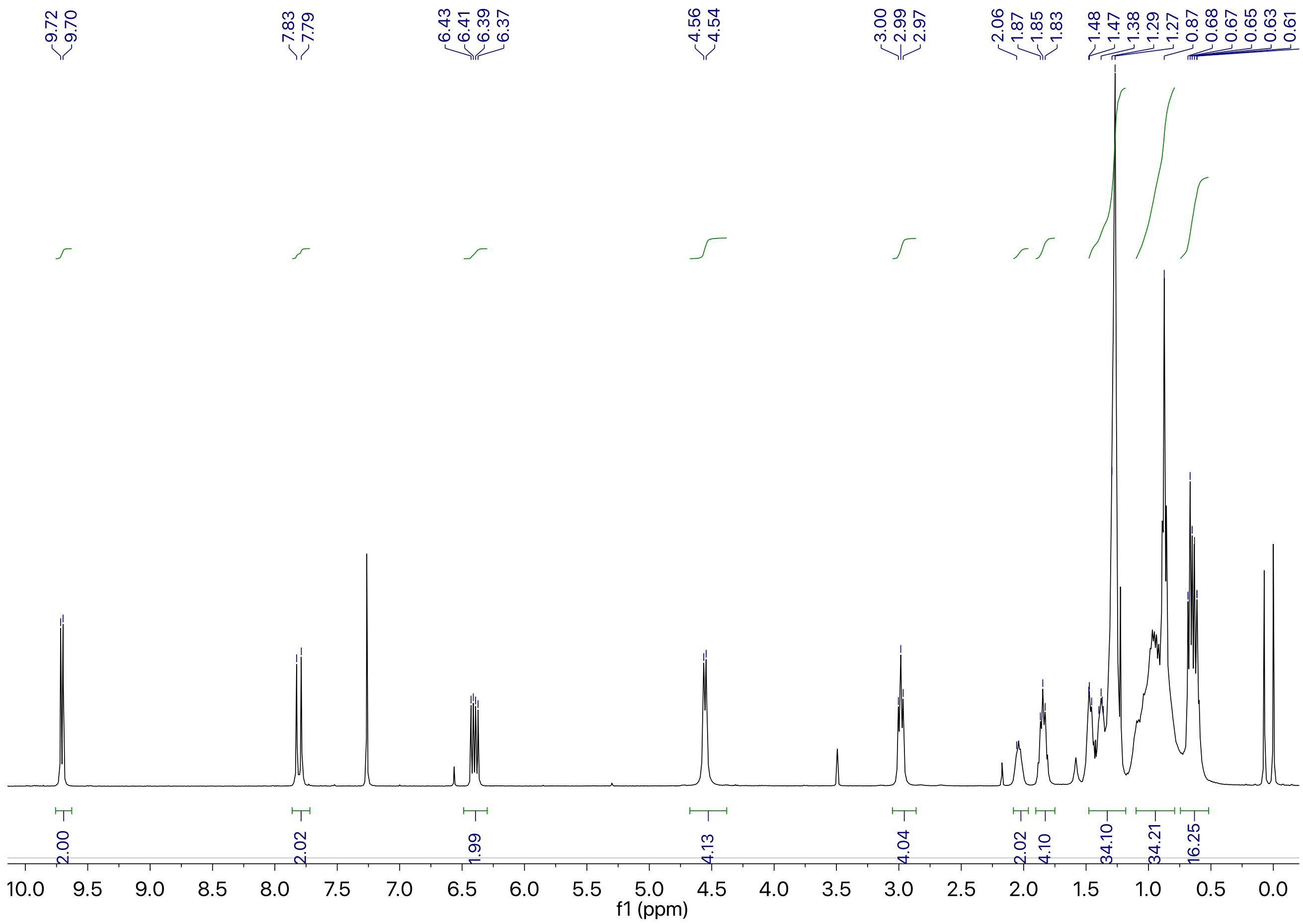

Supplementary Figure 20. ^{1}H-NMR spectrum of BTPSeV-CHO.

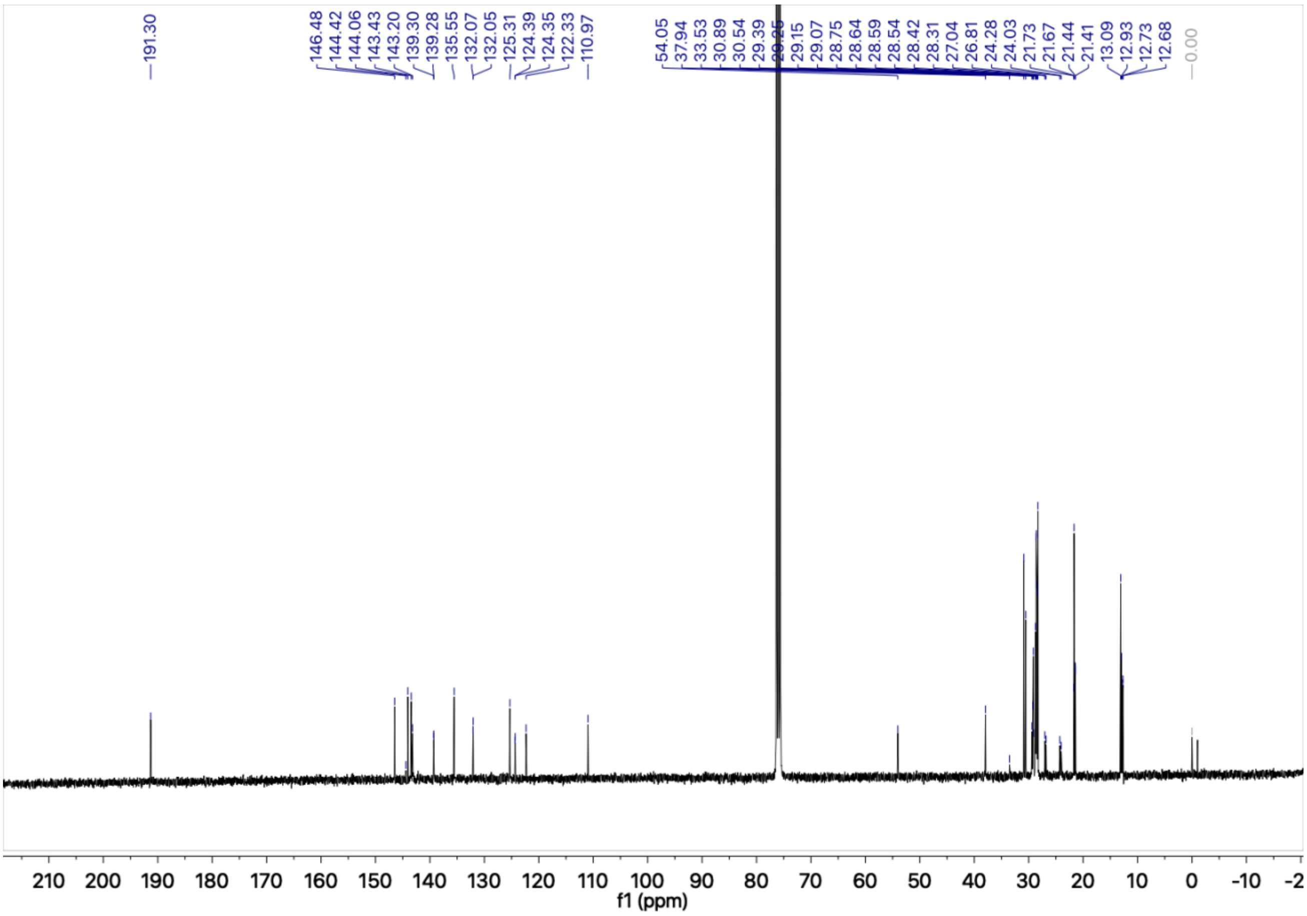

Supplementary Figure 21. ^{13}C-NMR spectrum of BTPSeV-CHO.

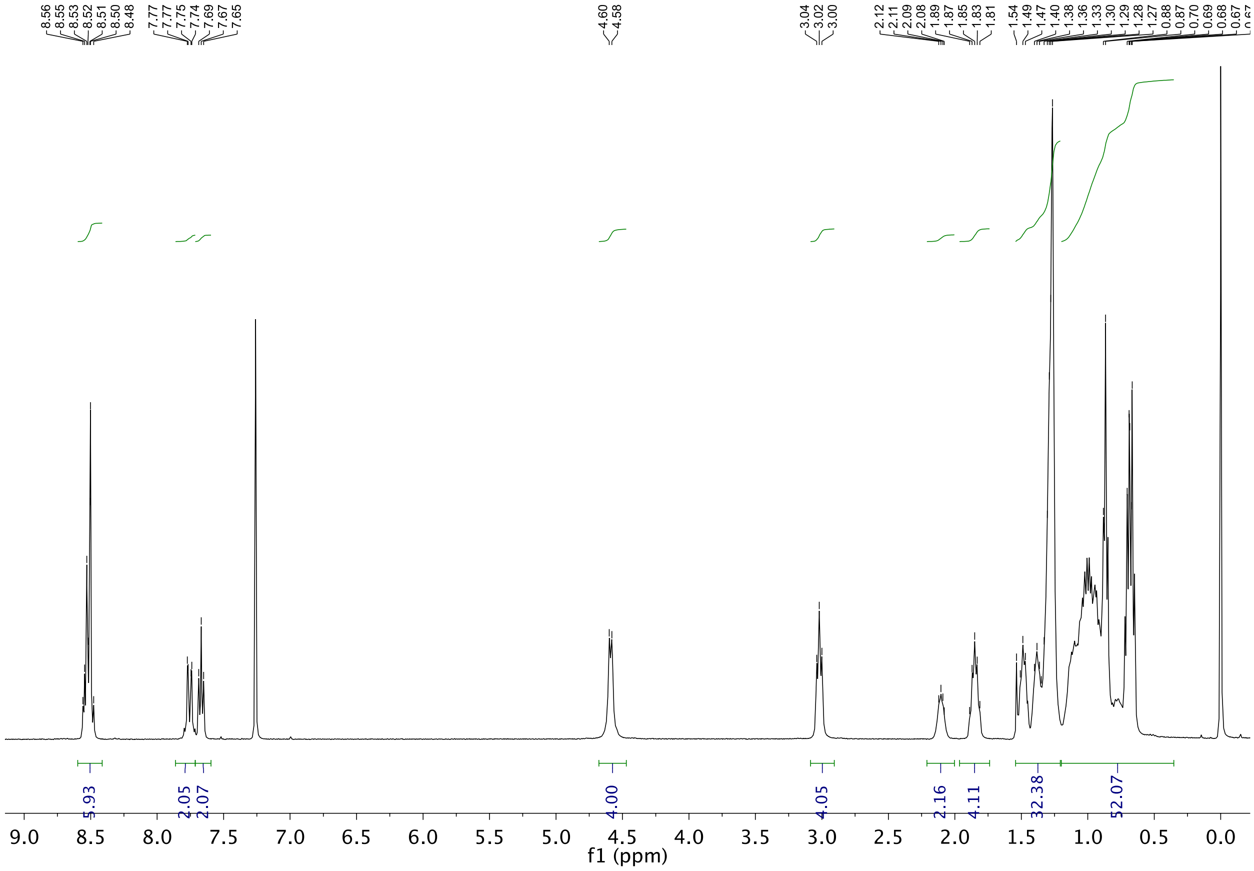

Supplementary Figure 22. ^{1}H-NMR spectrum of BTPSeV -4F.

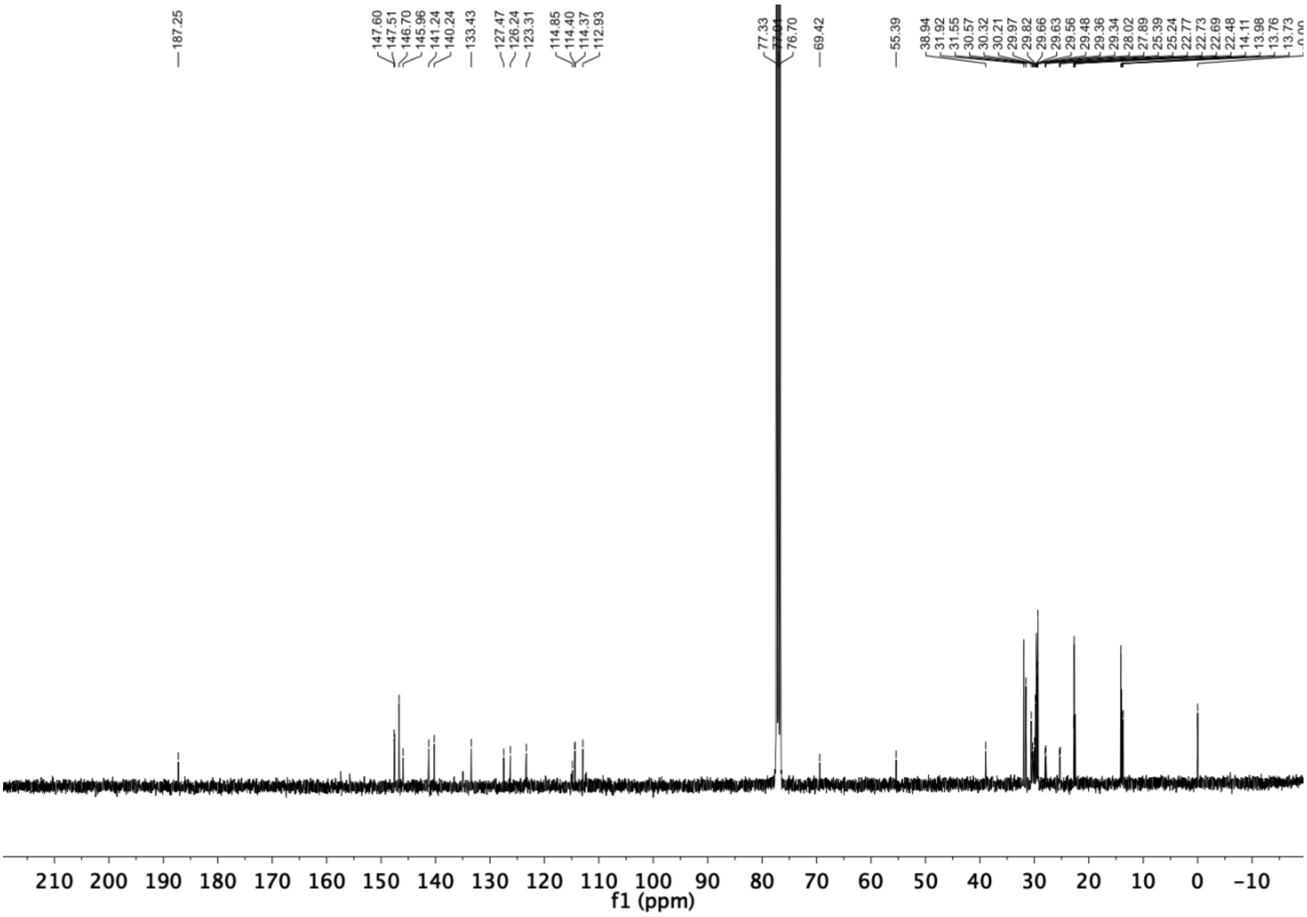

Supplementary Figure 23. ^{13}C-NMR spectrum of BTPSeV-4F.

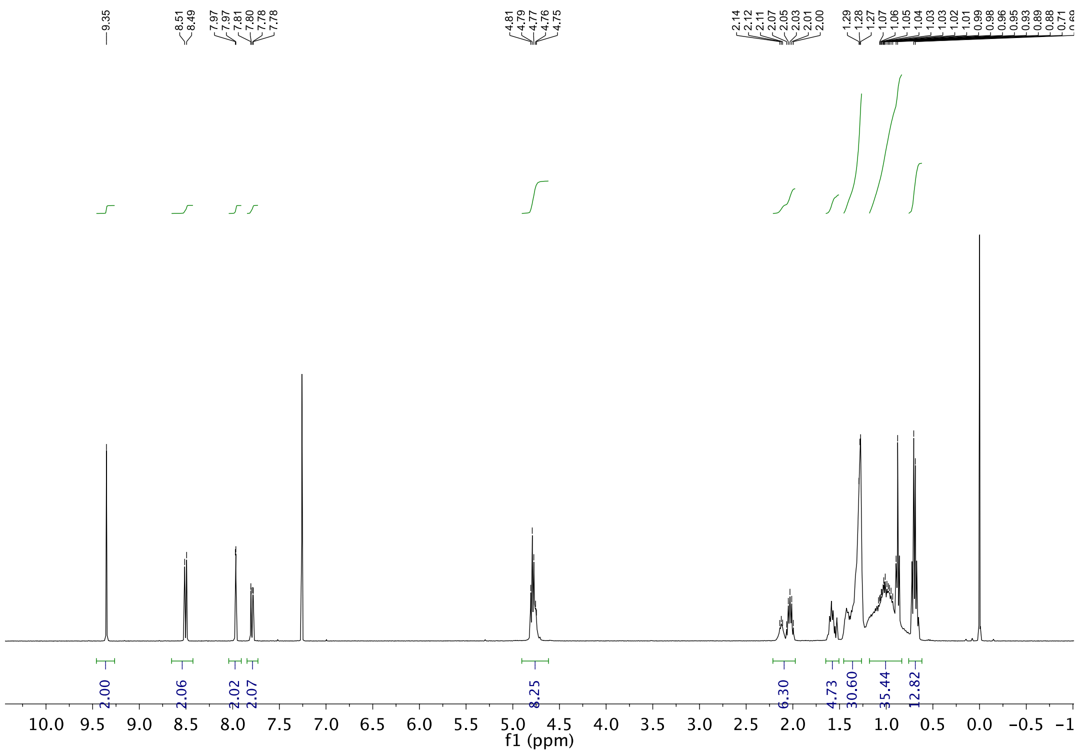

Supplementary Figure 24. ^{1}H-NMR spectrum of O1-Br.

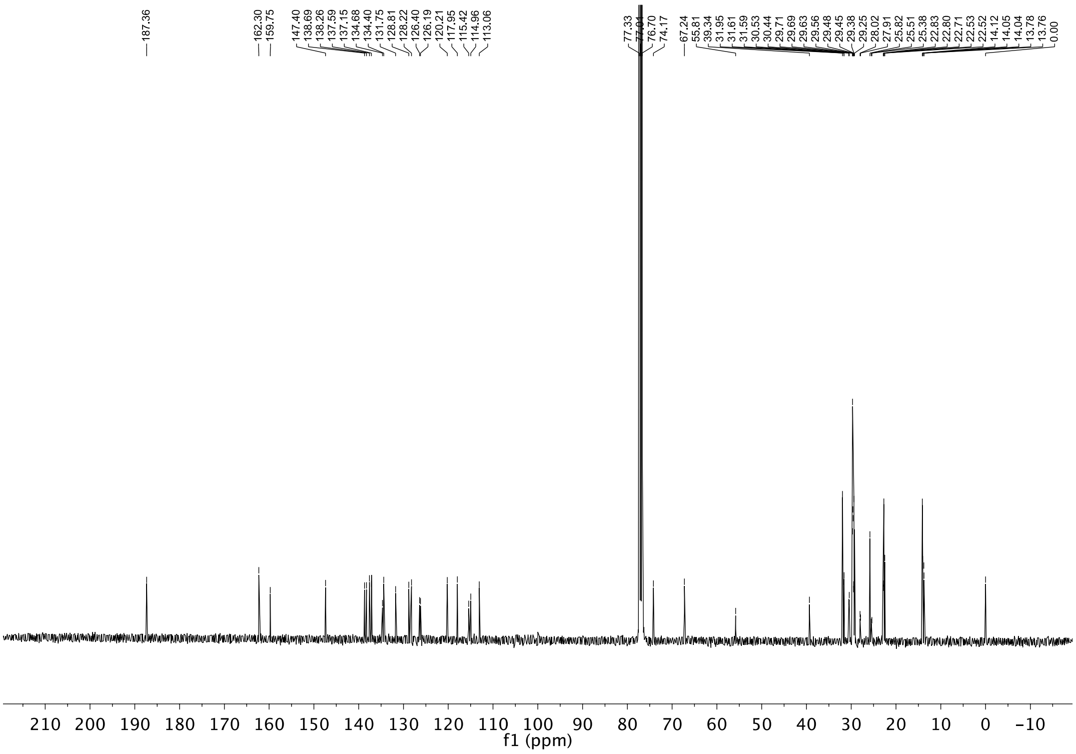

Supplementary Figure 25. ^{13}C-NMR spectrum of O1-Br.

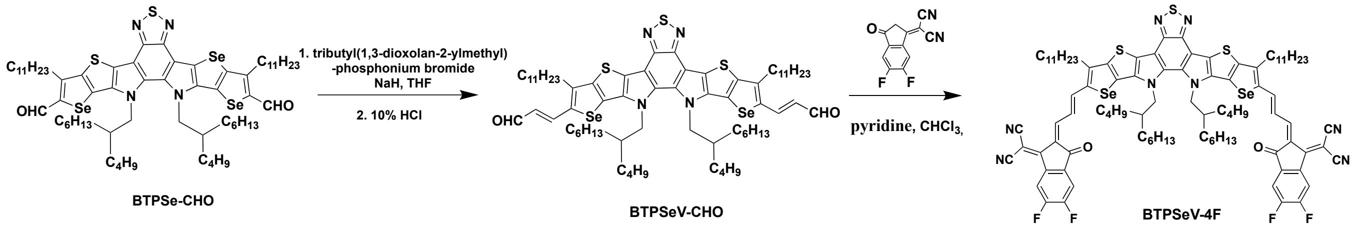

Supplementary Figure 26. Synthetic routes of BTPSeV-4F.

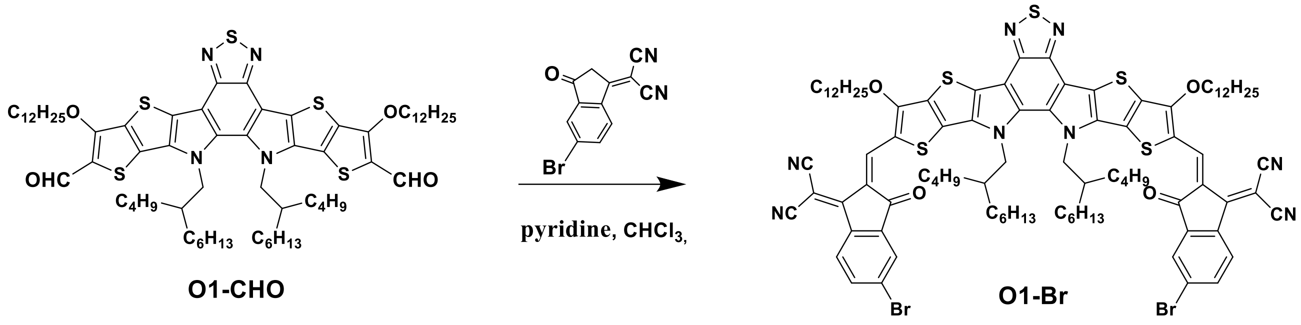

Supplementary Figure 27. Synthetic route of O1-Br.

SUPPLEMENTARY TABLES

Supplementary Table 1. Physicochemical properties and electronic energy levels of acceptors.

Acceptors	λ_{max}[a] (nm)	λ_{edge}[a] (nm)	E_g^{opt} [b] (eV)	E_{HOMO}[c] (eV)	E_{LUMO}[c] (eV)
O1-Br	697	786	1.58	-5.70	-3.76
BTPSV-4F	821	1021	1.21	-5.38	-3.96
BTPSeV-4F	853	1061	1.17	-5.36	-4.00

[a]Absorption of the films. [b] Optical bandgap calculated from the absorption edge of the films: E_g^{opt} = $1240/\lambda_{edge}$. [c] Energy levels calculated according to the equation $E_{LUMO/HOMO} = -e\,(E_{red/ox} + 4.36)$ (eV)

Supplementary Table 2. Photovoltaic parameters of the PSCs based on PTB7-Th:BTPSV-4F blend films with different processing conditions, under the illumination of AM1.5G, 100 mW cm^{-2}.

Donor:acceptor	Ratio	Additive (%)	Annealing (℃)	V_{oc} (V)	J_{sc} (mA cm^{-2})	FF (%)	PCE (%)
PTB7-Th:BTPSeV-4F	1:1.4	CN (0.5)	100	0.66	29.5	71.2	13.9
	1:1.6	CN (0.2)	100	0.66	27.9	69.6	12.8
		CN (0.5)	\	0.67	28.5	68.3	13.0
			90	0.66	29.7	70.5	13.8
			100	0.66	30.1	71.4	14.2
			110	0.66	29.3	71.0	13.7
			120	0.66	28.2	70.6	13.1
		CN (0.8)	100	0.66	28.7	72.2	13.7
	1:1.8	CN (0.5)	100	0.66	29.2	69.8	13.5

Supplementary Table 3. Charge carrier mobilities of the acceptors and their corresponding blend films.

Active layer	μ_e (10^{-4}cm^{2}V^{-1}s^{-1})	μ_h (10^{-4}cm^{2}V^{-1}s^{-1})	Active layer thickness (nm)
BTPSV-4F	4.2	/	70.0
BTPSeV-4F	3.5	/	72.2
PTB7-Th:BTPSV-4F	2.5	2.3	69.8/63.4
PTB7-Th:BTPSeV-4F	4.1	3.6	70.1/69.9

Supplementary Table 4. Energy loss analysis of the OSCs based on PTB7-Th:BTPSV-4F and PTB7-Th:BTPSeV-4F.

Devices	V_{oc} (V)	E_g^{pv} (eV)	qV_{oc}^{SQ} (eV)	ΔE_1 (eV)	EQE_{EL} (%)	ΔE_3 (eV)	ΔE_2 (eV)
PTB7-Th:BTPSV-4F	0.66	1.25	0.99	0.26	3.2×10^{-3}	0.27	0.05
PTB7-Th:BTPSeV-4F	0.66	1.22	0.96	0.26	7.4×10^{-3}	0.25	0.04

Supplementary Table 5. Summary of the GIWAXS parameters for the neat films.

Neat films	π-π stacking distance		π-π stacking coherence		Normalized Integrated Intensity
	q (Å^{-1})	d (Å)	Δq (Å^{-1})	CCL [010] Å	
BTPSV-4F	1.76	3.57	0.23	27	0.27
BTPSeV-4F	1.78	3.53	0.30	21	1.00

Supplementary Table 6. Summary of the GIWAXS parameters (of the donor) for the blend films with optimized conditions.

Blend films	π-π stacking distance		π-π stacking coherence		Normalized Integrated Intensity
	q (Å^{-1})	d (Å)	Δq (Å^{-1})	CCL [010] Å	
PTB7-Th: BTPSV-4F	1.70	3.71	0.26	24	1.40
PTB7-Th: BTPSeV-4F	1.72	3.66	0.43	15	1.00

Supplementary Table 7. Summary of the GIWAXS parameters (of the acceptors) for the blend films with optimized conditions.

Blend films	π-π stacking distance		π-π stacking coherence		Normalized Integrated Intensity
	q (Å^{-1})	d (Å)	Δq (Å^{-1})	CCL [010] Å	
PTB7-Th: BTPSV-4F	1.80	3.49	0.14	43	0.02
PTB7-Th: BTPSeV-4F	1.79	3.51	0.11	59	1.00

Supplementary Table 8. Photovoltaic parameters of the OSCs based on PM6:O1-Br blend films with different processing conditions, under the illumination of AM1.5G, 100 mW cm^{-2}.

Donor:acceptor	Weight ratio	Additive (vol%)	Annealing (℃)	V_{oc} (V)	J_{sc} (mA cm^{-2})	FF (%)	PCE (%)
PM6:O1-Br	1:1	CN (0.6)	100	1.04	19.5	73.5	14.9
	1:1.2	CN (0.3)	100	1.04	19.1	72.3	14.4
		CN (0.6)	\	1.05	18.7	71.1	14.0
			90	1.04	20.2	73.3	15.4
			100	1.04	20.0	74.4	15.5
			110	1.04	19.6	74.1	15.1
			120	1.04	19.2	72.6	14.5
		CN (0.9)	100	1.03	19.3	73.8	14.7
	1:1.4	CN (0.6)	100	1.04	19.5	74.0	15.0

Supplementary Table 9. Survey of the V_{oc} of the OSCs vs optical bandgaps of the acceptors used in the tandem OSCs reported in literatures.

Active layer	Optical bandgap (eV)	V_{oc} (V)	$E_{loss, onset}$ (eV)	J_{sc} (mA cm^{-2})	FF (%)	PCE (%)	Ref.
PBDB-T:F-M	1.65	0.98	0.67	14.56	71.0	10.08	1
PTB7-Th:NOBDT	1.39	0.77	0.62	19.16	70.0	10.26	1
PBDB-T:F-M	1.65	0.94	0.71	15.96	69.8	10.45	2
PTB7-Th:O6T4F:$PC_{71}BM$	1.26	0.69	0.57	27.6	69.7	13.29	2
PBDB-T:F-M	1.65	0.94	0.71	15.96	69.8	10.45	3
PBDB-T:NNBDT	1.43	0.86	0.57	20.07	69.7	12.03	3
PBDB-T:IT-M	1.63	0.91	0.72	16.3	71.4	11.0	4
PTB7-TH:FOIC:F8IC	1.33	0.72	0.61	21.9	69.1	11.1	4
PBDB-T/ITIC-Th3	1.63	0.93	0.7	15.9	70.8	10.9	5
PBDB-T/Y1	1.4	0.88	0.52	21.5	70.7	13.7	5
PBDB-T:ITIC	1.59	0.902	0.688	16.73	70.8	10.68	6
PTB7-Th:T2	1.3	0.67	0.63	22.65	0.66	10.1	6
DTDCPB:C70	1.77	0.9	0.87	16.2	67	9.8	7
PCE-10:BTCIC	1.39	0.69	0.7	22.1	70	10.7	7
PM6:SFT8-4F	1.58	0.98	0.6	16.4	67	12.1	8
PCE-10:BT-CIC:BEIT-4F	1.37	0.7	0.67	24.3	68	11.7	8
PM6:TfIF-4FIC	1.61	0.98	0.63	17.6	76	13.1	9
PTB7-Th:IEICO-4F	1.24	0.69	0.55	25.3	70	12.2	9
PM7:TfIF-4Cl	1.57	0.97	0.6	17.67	80	13.64	10
PCE10:CO*i*8DFIC:$PC_{71}BM$	1.26	0.69	0.57	27.39	74	14.05	10
PBDB-TF:ITCC	1.67	1.1	0.57	14,5	70.19	11.2	11
PBDB-TF:BTP-eC11	1.35	0.849	0.501	24.95	70.2	14.87	11

PM6:*m*-DTC-2F	1.61	0.99	0.62	17.4	68.3	12.1	[12]
PTB7-Th:BTPV-4F	1.21	0.67	0.54	28.9	69.3	13.4	[12]
PTQ10:*m*-DTC-2Cl	1.63	0.992	0.638	16.67	73.42	12.14	[13]
PTB7-Th:BTPV-4F-eC9	1.19	0.661	0.529	28.2	68.48	12.77	[13]
PBDB-T:F-M	1.65	0.99	0.66	70.0	15.52	10.75	[14]
PCE10:3TT-OCIC	1.29	0.69	0.6	68.9	27.58	13.13	[14]
PBDB-T:ITCC-M	1.68	1.03	0.65	14.8	66.3	10.1	[15]
PBDTTT-E-T:IEICO	1.36	0.823	0.537	18.8	66.7	10.3	[15]
PM6:O1-Br	1.59	1.04	0.55	20.0	74.4	15.5	**This work**
PTB7-Th:BTPSeV-4F	1.17	0.66	0.51	30.1	71.4	14.2	**This work**

Supplementary Table 10. Photovoltaic performance parameters of the tandem OSCs with different thicknesses of the sub-cells, under the illumination of AM1.5G, 100 mW cm^{-2}.

Thickness (nm)		V_{OC}	J_{SC}	FF	PCE
Front cell	Rear cell	(V)	(mA cm^{-2})	(%)	(%)
80	100	1.69	14.2	75.1	18.0
100	100	1.69	15.0	74.8	19.0
120	100	1.69	14.4	73.5	17.9
100	80	1.69	14.7	74.5	18.5
100	120	1.69	14.5	73.9	18.1

Supplementary References

1. Zhang Y*, et al.* Nonfullerene Tandem Organic Solar Cells with High Performance of 14.11%. *Adv. Mater.* **30**, 1707508 (2018).
2. Meng L*, et al.* Organic and solution-processed tandem solar cells with 17.3% efficiency. *Science* **361**, 1094-1098 (2018).
3. Meng L*, et al.* A Tandem Organic Solar Cell with PCE of 14.52% Employing Subcells with the Same Polymer Donor and Two Absorption Complementary Acceptors. *Adv. Mater.* **31**, 1804723 (2019).
4. Cheng P*, et al.* Efficient Tandem Organic Photovoltaics with Tunable Rear Sub-cells. *Joule* **3**, 432-442 (2019).
5. Cheng P*, et al.* Enabling High-Performance Tandem Organic Photovoltaic Cells by Balancing the Front and Rear Subcells. *Adv. Mater.* **32**, 2002315 (2020).
6. Chen F-X*, et al.* Near-Infrared Electron Acceptors with Fluorinated Regioisomeric Backbone for Highly Efficient Polymer Solar Cells. *Adv. Mater.* **30**, 1803769 (2018).
7. Che X, Li Y, Qu Y, Forrest SR. High fabrication yield organic tandem photovoltaics combining vacuum- and solution-processed subcells with 15% efficiency. *Nat. Energy* **3**, 422-427 (2018).
8. Huang X*, et al.* 15.9% organic tandem solar cell with extended near-infrared absorption. *Appl. Phy. Lett.* **116**, 153501 (2020).
9. Liu G*, et al.* 15% Efficiency Tandem Organic Solar Cell Based on a Novel Highly Efficient Wide-Bandgap Nonfullerene Acceptor with Low Energy Loss. *Adv. Energy Mater.* **9**, 1803657 (2019).
10. Liu G*, et al.* Tandem organic solar cells with 18.7% efficiency enabled by suppressing the charge recombination in front sub-cell. *Adv. Funct. Mater.* **31**, 2103283 (2021).
11. Wang J*, et al.* A Tandem Organic Photovoltaic Cell with 19.6% Efficiency Enabled by Light Distribution Control. *Adv. Mater.* **33**, 2102787 (2021).
12. Jia Z*, et al.* High performance tandem organic solar cells via a strongly infrared-absorbing narrow bandgap acceptor. *Nat. Commun.* **12**, 178 (2021).
13. Qin S*, et al.* Non-Halogenated-Solvent Processed and Additive-Free Tandem Organic Solar Cell with Efficiency Reaching 16.67%. *Adv. Func. Mater.* **31**, 2102361 (2021).

14. Gao H-H, *et al.* Achieving Both Enhanced Voltage and Current through Fine-Tuning Molecular Backbone and Morphology Control in Organic Solar Cells. *Adv. Energy Mater.* **9**, 1901024 (2019).

15. Cui Y, *et al.* Fine-tuned photoactive and interconnection layers for achieving over 13% efficiency in a fullerene-free tandem organic solar cell. *J. Am. Chem. Soc.* **139**, 7302-7309 (2017).

16. Gillett AJ, *et al.* The role of charge recombination to triplet excitons in organic solar cells. *Nature* **597**, 666-671 (2021).
